# Improvement of HEK293 Cell Growth by Adapting Hydrodynamic Stress and Predicting Cell Aggregate Size Distribution

**DOI:** 10.3390/bioengineering10040478

**Published:** 2023-04-16

**Authors:** Stefan Seidel, Rüdiger W. Maschke, Fruhar Mozaffari, Regine Eibl-Schindler, Dieter Eibl

**Affiliations:** Institute of Chemistry and Biotechnology, School of Life Sciences and Facility Management, ZHAW Zurich University of Applied Sciences, 8820 Wädenswil, Switzerland

**Keywords:** aggregate size distribution, biochemical engineering, CFD computational fluid dynamics, energy dissipation rate, fluid dynamic stress, HEK293 suspension culture, Kolmogorov length

## Abstract

HEK293 is a widely used cell line in the fields of research and industry. It is assumed that these cells are sensitive to hydrodynamic stress. The aim of this research was to use particle image velocimetry validated computational fluid dynamics (CFD) to determine the hydrodynamic stress in both shake flasks, with and without baffles, and in stirred Minifors 2 bioreactors to evaluate its effect on the growth and aggregate size distribution of HEK293 suspension cells. The HEK FreeStyle^TM^ 293-F cell line was cultivated in batch mode at different specific power inputs (from 63 $W\;m^{-3}$ to 451 $W\;m^{-3}$), whereby $\approx 60\;W\;m^{-3}$ corresponds to the upper limit, which is what has been typically described in published experiments. In addition to the specific growth rate and maximum viable cell density VCD${}_{\max}$, the cell size distribution over time and cluster size distribution were investigated. The VCD${}_{\max}$ of $(5.77\pm 0.02)\cdot 10^{6}$cells m$L^{-1}$ was reached at a specific power input of 233 $W\;m^{-3}$ and was 23.8% higher than the value obtained at 63 $W\;m^{-3}$ and 7.2% higher than the value obtained at 451 $W\;m^{-3}$. No significant change in the cell size distribution could be measured in the investigated range. It was shown that the cell cluster size distribution follows a strict geometric distribution whose free parameter *p* is linearly dependent on the mean Kolmogorov length scale. Based on the performed experiments, it has been shown that by using CFD-characterised bioreactors, the VCD${}_{\max}$ can be increased and the cell aggregate rate can be precisely controlled.

## 1. Introduction

HEK293 cells are derived from embryonic human kidney cells and were first isolated in the 1970s by Frank Graham [1]. Many subtypes and derivates have been established since and have been used for both research and biotechnological applications [2,3]. HEK293 cells are the second most used cells in cell biology and the second most used mammalian cells in biopharmaceutical production [4,5]. They are used as platforms for the expression of recombinant proteins due to their high transfection efficiency, flexibility, and human origin [6,7,8,9]. In addition, HEK293 cells are also used for the production of viral vectors and vaccines based on adenoviruses [7], retroviruses [10], lentiviruses [11], influenza viruses [12], or virus-like particles [13,14,15]. Furthermore, HEK293 cells are used in cancer research [16] and diagnostics [17,18]. Table 1 lists the products produced with HEK293 cells that have been approved by the U.S. Food and Drug Administration (FDA) and European Medicines Agency (EMA). The data indicate that HEK293 cells are primarily used for the commercial production of gene and chimeric antigen receptor (CAR) T cell therapeutics.

HEK293 cells can grow adherent (e.g., HEK293-T) or in suspension (e.g., HEK293-F). A detailed analysis of different HEK293 cell lines can be found in the studies by Malm et al. [19] and Tan et al. [3]. HEK293 cells growing in suspension typically have mean cell diameters ranging from 14 μm to 16 μm [20,21,22] and a typical maximum specific growth rate $\mu_{\max}$ ranging from 0.020 $h^{-1}$–0.029 $h^{-1}$ [23,24,25,26]. Jang et al. [24] were able to demonstrate comparable growth rates between adherent growing and in suspension growing HEK293 cells. The FreeStyle^TM^ HEK293-F cells used here are clones that have been adapted for growth in suspension. For such cells, cell densities of up to $100\cdot 10^{6}$ cells m$L^{-1}$ can be achieved in chemically defined media [27]. The cultivation of HEK293 suspension cells, similar to other mammalian cells, typically takes place in stirred stainless steel reactors with up to 100 L of working volume [28,29,30] or in wave-mixed bioreactors [30,31,32]. HEK293 cells tend to aggregate, which becomes problematic at higher cell densities and may limit large-scale production [33,34]. According to Liang Zhao et al. [33], the aggregation of these cells can be related to the Ca^2+^ and Mg^2+^ content in the medium, as well as to the agitation speed in the bioreactor.

**Table 1:** FDA- and EMA-approved biologics produced with HEK293 cells, extended from Tan et al. [3], Pulix et al. [35], Dumont et al. [36], and Walsh and Walsh [37].

**Product Name**	**Application**	**Producer**
Abecma®	CAR T therapy against multiple myeloma	Bristol-Myers Squibb ^a,b^
Alprolix®	Factor IX replacement against haemophilia B	Swedish Orphan Biovitrum ^a^/Sanofi ^b^
Breyanzi®	CAR T therapy against blood cancer	Bristol-Myers Squibb ^a,b^
Elocta®/ Eloctate®	Factor VIII–Fc fusion protein against haemophilia A	Swedish Orphan Biovitrum ^a^/Sanofi ^b^
Glybera®	Cell-based gene therapy against lipoprotein lipase deficiency	UniQure biopharma ^c^
Kymriah®	CAR T therapy against lymphoblastic leukaemia and lymphoma	Novartis ^a,b^
Luxturna®	Adeno-associated virus-based RPE65 gene therapy against Leber congenital amaurosis	Novartis ^a^/Spark Therapeutics ^b^
Nuwiq®/ Vihuma®	Recombinant anti-hemophilic factor VIII against haemophilia A	Octapharma ^a,b^
Strimvelis®	Cell-based gene therapy against severe combined immunodeficiency due to adenosine deaminase deficiency	Orchard Therapeutics ^a^
Trulicity®	Glucagon-like peptide-1 receptor linked to IgG against type 2 diabetes	Eli Lilly ^a,b^
Vaxzevria®	Adenovirus-based spike protein vaccine against COVID-19	AstraZeneca ^a^
Yescarta®	CAR T therapy against large B-cell lymphoma	Kite Pharma ^a,b^
Xigris®	Recombinant active protein C against sepsis	Eli Lilly ^c,d^
Zalmoxis®	Retrovirus-based gene therapy against leukaemia	MolMed ^c^
Zolgensma®	Adeno-associated vector housing the survival motor neuron against spinal muscular atrophy	Novartis ^a,b^
Zynteglo®	Lentivirus-based gene therapy against β-thalassemia	Bluebird bio ^c^

^a^ Approved by EMA; ^b^ approved by FDA; ^c^ withdrawn by EMA; ^d^ withdrawn by FDA; CAR: chimeric antigen receptor; IgG: immunoglobulin G.

The amount of cell and gene therapeutics will greatly increase in the next few years for both experimental and approved biopharmaceuticals [37]. This particularly applies to CAR T cell therapeutics [37]. As shown in Table 1, these are successfully produced using HEK293 cells. In order to make the production of such therapeutics efficient, the upstream process must be understood. In this context, the process engineering characterisation of bioreactors helps to achieve this understanding [38,39,40]. The specific power input and hydrodynamic stress are among the most important process parameters and often serve as scale-up criteria [41]. Through optimal specific power input, the cell density can be increased; thus, under certain circumstances, the production of cell and gene therapeutics can also be made more efficient. The specific power input can be determined for stirred bioreactors and shake flasks by means of experimental measurement of the torque [42,43]. An alternative to experimental investigations is offered by computational fluid dynamics (CFD). With this approach, not only can average values such as the specific power input be determined, but it can also investigate spatially and temporally resolved values. For example, Seidel et al. [40] were able to carry out time-resolved investigations of the volume-related Kolmogorov length size distribution in a wave-mixed CELL-tainer bioreactor and thus estimate whether potentially harmful hydrodynamic stress occurs for mammalian cells.

The investigations described below deal with the mass propagation of HEK293 suspension cells up to the bench-top scale. The hypothesis is that through CFD simulations, it is possible to not only control the hydrodynamic stress but also the cell aggregation rate, culminating in an improvement of HEK293 cell growth.

## 2. Materials and Methods

**Figure 1:**
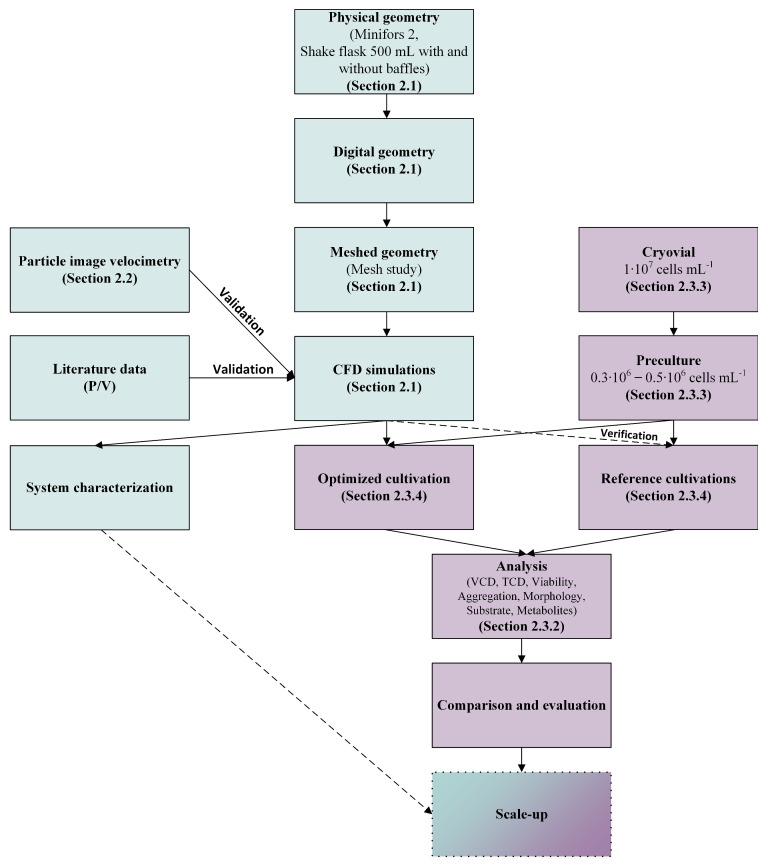
Graphical representation of the experiments carried out; the most important steps and the section in which the methods are described in detail are listed. Teal represents procedural steps and violet represents those with a focus on cell culture technology. The dot-framed step scale-up is not part of this study. However, the information gained here serves as a basis for a scale-up strategy.

In order to verify our hypothesis, a number of investigations were carried out, consisting of process engineering investigations (marked in teal in Figure 1) such as CFD and particle image velocimetry (PIV), as well as cell culture experiments (marked in violet in Figure 1). A summary of the methods used is shown in Figure 1 and will be described in detail in the following sections.

### 2.1. Computational Fluid Dynamics

**Figure 2:**
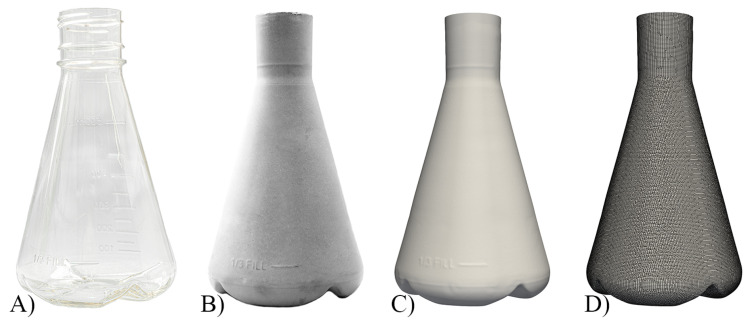
Process steps from the single-use shake flasks to computational mesh. (**A**) The 500 mL baffled shake flask. (**B**) Model cast with levelling compound. (**C**) Scanned and smoothed computer model. (**D**) Computational mesh used for the CFD simulations.

CFD investigations were carried out using the stirred Minifors 2 6 L bioreactor (Infors AG, Bottmingen, Switzerland) in the cell culture version (3-blade segment stirrer, no baffles, ring sparger) as well as 500 mL unbaffled and 500 mL baffled Erlenmeyer shake flasks from Corning Inc. (Corning, NY, USA). The geometry of the Minifors 2 was measured and digitised using Inventor Professional 2023 software (Autodesk Inc., San Rafael, CA, USA). The geometry of the two shake flasks was 3D-scanned; for this purpose, the method described by Seidel et al. [40] was used. The shake flasks (Figure 2A) were filled with levelling compound (Fliesst & Fertig schnell, Lugato GmbH & Co. KG, Barsbüttel, Germany). The only change was that hardening took place at room temperature. The shake flasks were then destructively removed, and the negative (Figure 2B) was scanned using an Einscan Pro 3D scanner (Shining 3D Tech. Co. Ltd., Hangzhou, China). The scan was reverse-engineered using EXScanPro and Blender 3.2 software [44]. In this process, the floor levelling compound and the 3D scanner are capable of mapping the volume scale on the inside of the shaking flask itself (Figure 2C). For all three geometries, the computational mesh was created using the blockMesh and SnappyHexMesh utilities from OpenFOAM version 10 (OpenFOAM software, The OpenFOAM Foundation Ltd, London, UK) (Figure 2D). The choice of computational mesh was determined by means of a qualitative and a quantitative mesh study, respectively (Section 3.3).

The simulations involving the stirred bioreactor were carried out as turbulent, single-phase, and steady-state simulations. The resulting Reynolds-Averaged Navier–Stokes (RANS) and continuity equations correspond to the Equation (1) and (2).

(1) $$\frac{\partial \vec{v}}{\partial t}+\nabla \;\cdot \;\left(\vec{v}\vec{v}\right)-\nabla \;\cdot \;\nu_{\mathrm{eff}}\nabla \vec{v}=-\frac{1}{\rho_{w}}\nabla p_{p}+\nabla \;\cdot \;S_{ij}$$

(2) $$\nabla \;\cdot \;\vec{v}=0$$

Here, $\vec{v}$ corresponds to the mean velocity vector and $p_{p}$ corresponds to the mean pressure, whereby the fluctuating values are approximated. $\nu_{\mathrm{eff}}$ corresponds to the sum of the turbulent eddy viscosity $\nu_{T}$ and the viscosity of the fluid $\nu_{w}$, *t* corresponds to the time, and $\rho_{w}$ corresponds to the density of the fluid. The Reynolds stress tensor $S_{ij}$ is determined by the turbulence model (Equation (3)). Here, *k* corresponds to the turbulent kinetic energy and *I* to the second order identity tensor.

(3) $$S_{ij}=\nu_{T}\left(\nabla \vec{v}+{\left(\nabla \vec{v}\right)}^{T}\right)-\frac{2}{3}\;\cdot \;\rho_{w}\;\cdot \;k\;\cdot \;I$$

The turbulence model used here is the *k*-ω shear stress transport (SST) model of Menter [45] (ω corresponds to the turbulent specific dissipation rate), as it can also be directly used as a model for low Reynolds numbers (a detailed derivation can be found in Appendix B). The rotation of the stirrer was handled by the multiple reference frame (MRF) approach as it is a steady-state analysis [46,47,48]. The relatively low speeds and axial pumping (less vortex formation compared with radial pumping stirrers) allow for the assumption of a non-free surface. This assumption was visually verified on the bioreactor. Therefore, a symmetry plane was used as boundary conditions for the liquid surface, as is usual for single-phase stirred bioreactor simulations [48,49,50]. A no-slip wall boundary condition was used for the bioreactor wall, stirrer, and internals [46,47]. The OpenFOAM solver simpleFOAM was employed, which uses the Semi-Implicit Method for Pressure-Linked Equations (SIMPLE) algorithm for the pressure-velocity coupling [51]. As a convergence criterion, an undershooting of the residuals of $10^{-5}$ was used.

Unlike the stirred bioreactor, the shake flasks cannot be assumed to be stationary, nor can the free surface be neglected. For these simulations, the volume of fluid (VOF) approach was used, in which a mixed fluid, with properties, is calculated (Equations (2), (4) and (5)) [52].

(4) $$\frac{\partial \rho \vec{v}}{\partial t}+\nabla \;\cdot \;\left(\rho \vec{v}\vec{v}\right)=-\nabla p+\rho \;\cdot \;\vec{g}+\nabla \;\cdot \;\nu_{\mathrm{eff}}\left(\nabla \vec{v}+{\left(\nabla \vec{v}\right)}^{T}\right)+\vec{F}$$

(5) $$\vec{F}=\sigma_{\mathrm{wa}}\;\cdot \;\kappa \;\cdot \;\nabla \alpha_{w}$$

The gravitational acceleration is represented by $\vec{g}$. The surface tension force $\vec{F}$ corresponds to the product of the surface tension $\sigma_{\mathrm{wa}}$, the local interfacial curvature κ (Equation (6)), and the gradient of the liquid phase fraction $\alpha_{w}$ [53,54]. The density ρ and kinematic viscosity ν of the fluids are weighted according to their phase fraction $\alpha_{i}$, whereby the two phases water $\alpha_{w}$ and air $\alpha_{a}$ occur in the system examined here (Equations (7) and (8)).

(6) $$\kappa =\nabla \;\cdot \;\left(\frac{\nabla \alpha_{w}}{|\alpha_{w}|}\right)$$

(7) $$\chi =\sum \chi_{i}\alpha_{i},\;\chi \in \left[\rho ,\nu \right]$$

(8) $$\sum \alpha_{i}=1\;\forall \alpha_{i},\left\{\alpha_{i}|0\leq \alpha_{i}\leq 1\right\}$$

As with the stirred bioreactor, the *k*-ω-SST model was utilised for turbulence modelling. Corning shake flasks are composed of polycarbonate. This was taken into account in the simulation via the contact angle $\theta_{\mathrm{pc}}$ of 83 ${}^{\circ }$ [55] and was therefore a constant contact angle boundary condition for the whole system. The contact angle $\theta_{\mathrm{pc}}$ influences the surface normal vector $\vec{n}$, which then influences the local curvature κ of the surface near the bioreactor wall (Equation (9)) [54]. $\vec{n_{n}}$ corresponds to the unit vector in the normal direction to the wall and $\vec{n_{t}}$ to the unit vector in the tangential direction.

(9) $$\vec{n}=\vec{n_{n}}\;\cdot \;\cos\left(\theta_{\mathrm{pc}}\right)+\vec{n_{t}}\;\cdot \;\cos\left(\theta_{\mathrm{pc}}\right)$$

The simulations of the shake flasks were performed as in Seidel et al. [40]. For this purpose, the solver interFOAM was used, which uses the PIMPLE algorithm for the pressure-velocity coupling (combination of the Pressure Implicit with Splitting of Operator (PISO) and the SIMPLE algorithm). The Courant-Friedrichs-Lewy number was kept below 0.9 and the piece-wise linear interface calculation (PLIC) algorithm was used for accurate free surface reconstruction [56,57,58,59].

All simulations were performed at a temperature of 310.15 K, which corresponds to the cultivation temperature of HEK293 cells (Section 2.3). At this temperature, the density of water ($\rho_{w}$) corresponds to 993.37 kg/$m^{3}$ and that of air ($\rho_{a}$) to 1.138 kg/$m^{3}$. The kinematic viscosity is $0.6959\cdot 10^{-6}$ m${}^{2}$/s for water ($\nu_{w}$) and $16.64\cdot 10^{-6}$ m${}^{2}$/s for air ($\nu_{a}$) [60]. The surface tension $\sigma_{\mathrm{wa}}$, which was relevant for the shake flask simulations, was $71.968\cdot 10^{-3}$ N/m [60]. The simulations were performed in parallel on the high-performance computing system described in Seidel and Eibl [61]. Paraview 5.10.0 and Python 3.10 software were used for post-processing [62,63]. To determine the discretisation error, a mesh study was carried out for both the shake flasks and the stirred bioreactor.

### 2.2. Particle Image Velocimetry

**Figure 3:**
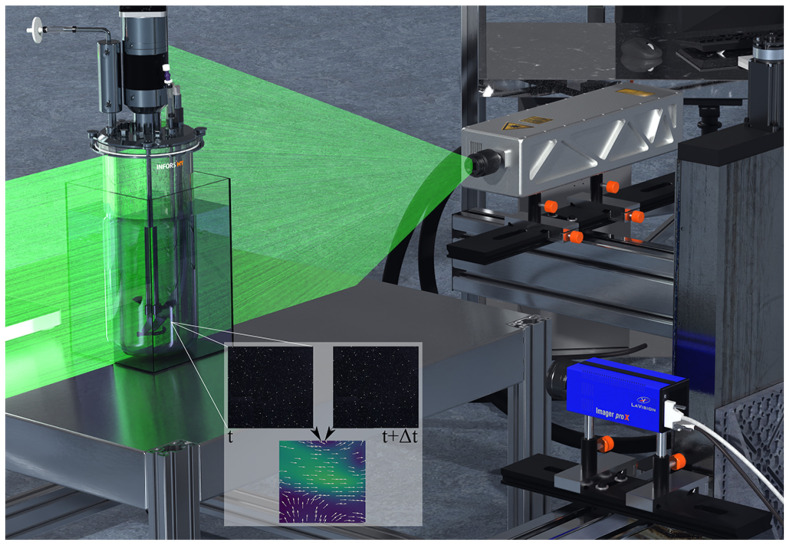
The figure shows a computer generated image (CGI) of the PIV system used. The investigated stirred bioreactor is placed in the centre of the measuring system. To minimise light refraction at the curved bioreactor wall, the bioreactor was placed in a rectangular, water-filled container. The bioreactor internals were coated in black to minimise reflection. At the top right of the figure, the double-pulse Nd:YAG laser can be seen as it emits a light pulse with a wavelength of 532 nm (green light). With the high-sensitivity camera at the bottom right, which is aligned at a 90${}^{\circ }$ angle to the light field, two images are recorded with a Δt time interval. The recorded images are shown in the centre of the figure for the time *t* and t+Δt. For each position, 1000 double images were evaluated and assembled into a two-dimensional vector field by means of cross-correlation.

For the validation of the CFD simulations, the velocity profile in the Minifors 2 bioreactor was measured using 2D-2C PIV (Figure 3). The results were then compared with the velocity profile calculated by CFD. The light source was a 145 mJ Bernoulli 145-15 PIV (Litron Lasers Ltd, Rugby, UK) double-pulse neodymium-doped yttrium aluminium garnet (Nd:YAG) laser. The light pulses, which had a wavelength of 532 nm, lasted 10 ns, and the width of the laser field was 1 mm. For the recording, a high-sensitivity 14-bit charge-coupled device (CCD) camera (Imager Pro X 4M with 2048 pixel × 2048 pixel) with a 50 mm fixed focal length lens was used (Nikkor Lens 50 mm, f/1.8D, Nikon Corporation, Tokyo, Japan). To measure the complete bioreactor, a laser and camera were mounted on a 3-axis linear translation stage from isel Germany AG (Eichenzell, Germany). According to camera position, 1000 double images were taken with a field of view of 45 mm × 45 mm. The images were captured using an external trigger laser (WL12L-2B530, Sick AG, Waldkirch, Germany) to keep the stirrer position constant. Fluorescent rhodamine B-coated poly(methyl methacrylate) (PMMA) tracer particles with a size of 20 μm to 50 μm were used ($\rho_{\mathrm{PMMA}}=1190\;kg/m^{3}$). To minimise reflections, all bioreactor internals were sprayed black, and to minimise light refraction at the curved bioreactor, the bioreactor was placed in a rectangular water-filled container [64]. Acquisition and image processing was carried out using DaVis 10.2.1 software (LaVision GmbH, Göttingen, Germany) by cross-correlation using sum-of-correlation with 6 multi-pass steps. The images were smoothed using a 3 pixel × 3 pixel Gaussian filter.

### 2.3. Cultivation

#### 2.3.1. Cell Line and Medium

The experiments were performed using HEK FreeStyle^TM^ 293-F suspension cells (Thermo Fisher Scientific, Waltham, MA, USA, [65]), a descendant of the HEK293-F cell line [66]. For the inoculum production and batch experiments, the chemically defined, animal origin–free, and protein-free FreeStyle^TM^ 293 medium (Gibco Thermo Fisher, Waltham, MA, USA) was used. In addition, the medium contains L-alanyl-L-glutamine (GlutaMAX^TM^), which is a stabilised, ready-to-use form of L-glutamine.

#### 2.3.2. Analytics

**Figure A1:**
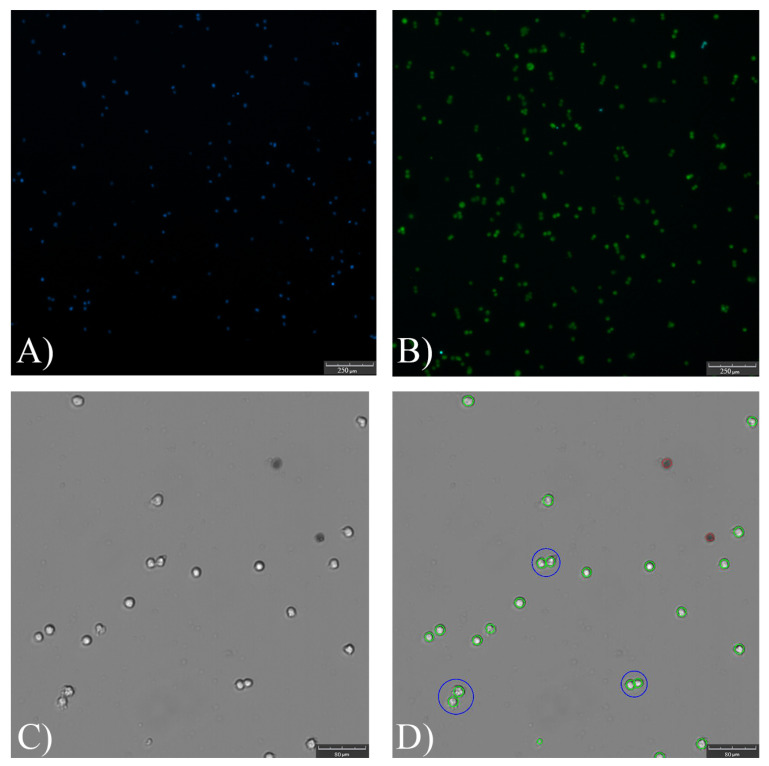
Viability analysis of HEK293 suspension cells cultivated in a baffled 500 mL shake flask. (**A**) First analysis step of the NucleoCounter Viability and Cell Count—Aggregated Cells Assay. Cells were disrupted with Solution 10. Blue shows dead cells stained with DAPI. (**B**) Second analysis step of the NucleoCounter Viability and Cell Count—Aggregated Cells Assay. Cells were not disrupted. Blue shows dead cells stained with DAPI and green shows cells where the nuclei were stained with acridine orange. (**C**) Raw image from the CedexHiRes analyser, which stained cells with trypan blue (1 of 10 evaluated images per sample). (**D**) Automatic evaluation of image (**C**) cells marked in green correspond to living cells, cells marked in red correspond to dead cells, and cell groups circled in blue correspond to aggregates. Scale bars for (**A**,**B**) are 250 μm and are 80 μm for (**C**,**D**).

Cell-specific parameters such as viable cell density (VCD), total cell density (TCD), viability, and cell diameter were measured daily using a CedexHiRes analyser (Roche Diagnostics GmbH, Basel, Switzerland) and a NucleoCounter NC-200 (Chemometec, Allerod, Denmark). The measurement in the NucleoCounter is based on the dyes DAPI (4’,6-Diamidino-2-phenylindol) and acridine orange, which stain the cell nuclei of HEK293 cells. DAPI can only penetrate the cell membranes of intact cells very slowly and therefore primarily stains dead cells (or cells with damaged cell membranes), while acridine orange stains both living and dead cells. However, exact measurement is problematic in larger aggregates where the cell nuclei can overlap, so the Viability and Cell Count—Aggregated Cells Assay was used here. In this assay, a double measurement is performed: by adding Solution 10 (lysis buffer of tensides and organic acid), the cells are killed and the aggregates are disrupted, allowing the cell count to be determined more accurately (Appendix A Figure A1A). Viability is determined without the addition of Solution 10 (Appendix A Figure A1B). Because the NucleoCounter NC-200 measures cell nuclei, morphological data are limited. For a more detailed investigation of cell size, form factor, and aggregation rate, the CedexHiRes analyser was used. This is based on automated microscope imaging combined with trypan blue staining for cells with damaged cell membranes. Cells and aggregates with diameters ranging from 2 μm to 40 μm can be measured at a resolution of 0.8 μm/pixel, with an average of 10 individual images (Figure A1C,D).

The concentration of metabolites (lactate and ammonia) and substrates (glucose and L-alanyl-L-glutamine) were determined using the CedexBio analyser (Roche Diagnostics GmbH). Glucose concentration was determined by the hexokinase-driven phosphorylation of glucose to glucose-6-phosphate. Subsequently, reduced nicotinamide adenine dinucleotide phosphate (NADPH) is formed by the oxidation of NADP+, the formation of which is photometrically measured at 340 nm. L-alanyl-L-glutamine is first hydrolysed enzymatically via amino acid arylamidase to glutamine and alanine. Subsequently, glutamine is deaminated by glutaminase to glutamate, which is oxidised by glutamate oxidase to α-ketoglutarate, ammonium, and hydrogen peroxide. The resulting hydrogen peroxide, together with 4-amino antipyrine and N-ethyl-N-(2-hydroxy-3-sulfopropyl)-m-toluodine, is oxidised by peroxidase to water and a chromogen, the concentration of which is determined. In the lactate measurement, cleavage of L-lactate occurs by lactate oxidase to pyruvate and hydrogen peroxide, which is determined analogously to the description in the L-alanyl-L-glutamine measurement. In the ammonium measurement, a reaction with 2-oxoglutarate and NADPH occurs to form glutamate, NADP+, and water. The decrease in NADPH is determined analogously to the glucose measurement.

**Figure A2:**
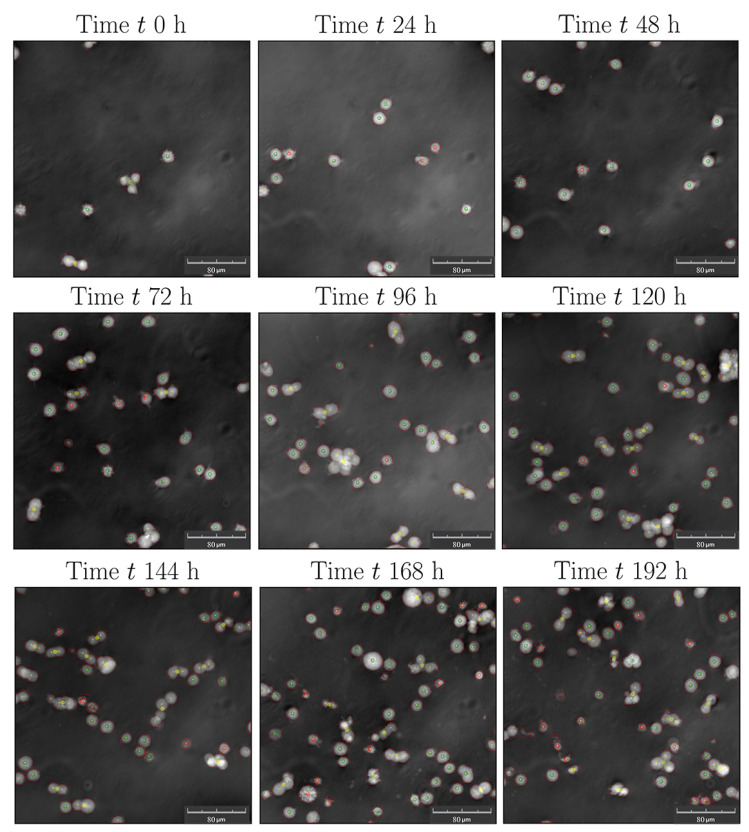
Phase-shift microscopy images from the iLine F analyser (scale bars are 80 μm). The images (only one focal plane is shown here) serve as the basis for determining the optical height. The image series shows different times of Minifors 2 cultivation (N=180 rpm, V=4 L) and corresponds to the optical heights from Figure 11.

**Figure A3:**
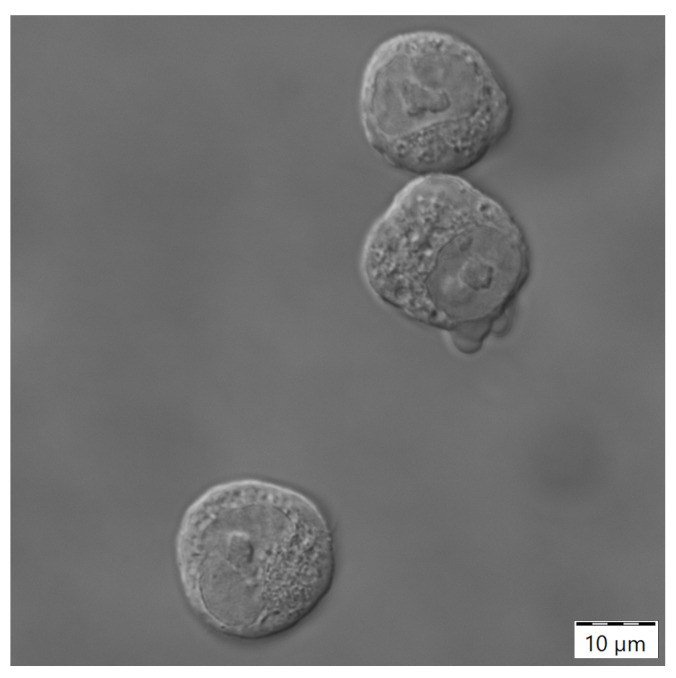
Differential interference microscopy image of HEK293 suspension cells cultivated in a baffled 500 mL shake flask (scale bar is 10 μm).

Continuous monitoring of VCD, TCD, viability, cell morphology, and aggregation was also performed using an iLine F analyser (Ovizio Imaging Systems NV/SA, Uccle, Belgium). The iLine F analyser is a non-invasive in-line cell counting instrument based on the principles of quantitative phase microscopy and digital holography. The wavefront of the light, which is affected by the cells to be measured, is recorded by a CCD camera and, together with a phase-shifted image, numerically assembled into a three-dimensional structure (Figure A2). Cells with diameters ranging from 2 μm to 100 μm and cell concentrations ranging from $1\cdot 10^{5}$ cells m$L^{-1}$ to more than $2\cdot 10^{7}$ cells m$L^{-1}$ can be measured. In addition to counting live and dead cells, a variety of morphological parameters such as size, shape, and thickness can be analysed. The iLine F analyser includes the main components, the Ovizio reader, the disposable BioConnect probe, and a pump connected to the BioConnect probe. The BioConnect probe consists of two parts. Firstly, there is a sterile, disposable pump and a fluidic system that is integrated into the bioreactor and transfers the cells to the Ovizio reader. The second part is the pump engine, which is not disposable. The measurements run dye-free automatically and via continuous real-time monitoring with no manual sampling required. The total magnification of the Ovizio reader is 22.2×, and the horizontal resolution is 1.5 μm. The measurements were reported using the software OsOne 7.3.0 [67]. Further information on quantitative phase microscopy can be found in Kim [68]. In addition, the cells were also viewed offline using differential interference microscopy (Figure A3). Images were taken with a fully automated IX83 inverted microscope and a UPlanSApo 100x/1.4 oil *∞*/0.17/FN26.5 objective (both Olympus Life Science, Waltham, MA, USA). To measure the osmolality of the FreeStyle^TM^ 293 medium, the semi-micro freezing point osmometer K-7400S was used (KNAUER Wissenschaftliche Geräte GmbH, Berlin, Germany).

#### 2.3.3. Inoculum Production

For the inoculum production of 125 mL and 250 mL, unbaffled disposable shake flasks (Corning Inc.) were used. In the first step, cryovials from the working cell bank with a VCD of $1\cdot 10^{7}$ cells m$L^{-1}$ were thawed, and the cells were transferred into a 125 mL shake flask with 30 mL prewarmed FreeStyle^TM^ 293 medium. Inoculum production took seven days each time, with passaging to a VCD ranging from 0.3 $\cdot \;10^{6}\;\mathrm{cells}$ m$L^{-1}$ to 0.5 $\cdot \;10^{6}\;\mathrm{cells}$ m$L^{-1}$ occurring every second or third day. The shake flasks were incubated in a Multitron shaker (Infors AG) at a temperature of T=310.15 K, a shaking speed of N=100rpm, a shaking amplitude of $d_{0}=50\;mm$, a ${\mathrm{CO}}_{2}$ concentration of $c_{CO_{2}}=8\%$, and a relative humidity of RH=80%.

#### 2.3.4. Cultivation Systems and Cultivation

The cultivation of the HEK293 cells was performed in a Minifors 2 6 L cell culture version (Infors AG) with a working volume of 4 L. In addition, the experiments were carried out in baffled and unbaffled 500 mL shake flasks (Corning Inc.) with a working volume of 160 mL.

Cultivations in the baffled and unbaffled shake flasks were performed as quintuplicates. The ten shake flasks were inoculated with a VCD of $0.3\cdot 10^{6}$ cells m$L^{-1}$. For all shake flasks, the same inoculum was used. The inoculum had a viability of >98%, and the cells were in passage 15. The shake flasks were incubated in the incubation shaker at a rotating speed of 130 rpm ($d_{0}=50\;mm$). The other parameters were set to be the same as for the inoculum production. At viabilities below 70%, the experiments were terminated. The cultivations in the Minifors 2 bioreactor were carried out as a double determination. The stirred bioreactor was inoculated with the same cell density as the shake flasks and terminated at the same conditions. The pH value was kept at 7.1 using CO_2_ as the acid with no base addition. Dissolved oxygen concentration (DO) was kept above 40% with a constant headspace aeration rate of 0.1 vvm air and sparging with O_2_ when necessary. The temperature was kept at 310.15 K. The Minifors 2 was equipped with a 3-blade segment impeller with a diameter of 85 mm. To investigate cell growth and viability under different hydrodynamic stress conditions, the stirrer speed was set to 180 rpm, 275 rpm, and 350 rpm. A stirrer speed of 180 rpm in the stirred bioreactor and 130 rpm in the shaking incubator correspond to specific power inputs where HEK293 cells are typically cultured [2,69,70,71,72,73].

## 3. Results and Discussion

### 3.1. CFD for Shake Flasks

In order to estimate the spatial discretisation error on the one hand and to perform economic CFD simulations on the other hand, a mesh study was carried out. This was quantitatively performed using the grid convergence index (GCI) method, which corresponds to the Richardson extrapolation with a safety factor of $F_{s}$ [74,75,76]. This method is considered to be the best practice and is recommended by the OECD [77]. A detailed explanation of the procedure can be found in [78,79,80]. Five computational meshes with 0.28 $\cdot \;10^{6}\;\mathrm{cells}$ to 2.09 $\cdot \;10^{6}\;\mathrm{cells}$ were created for the studies, resulting in 3 GCI cases (Table 2). In each case, the mesh refinement factor *r* ranged between 1.1 and 1.3 [74]. A safety factor $F_{s}$ of 1.25 was used for the investigations [78,81,82]. The investigated criterion chosen was the mean Kolmogorov length, which occurs during one complete shaking period. Table 2 shows the results for the investigations of the baffled shake flask. As can be deduced from the quotients $\frac{{\mathrm{GCI}}_{i+2,i+1}}{r^{p_{a}}{\mathrm{GCI}}_{i+1,i}}$, the solutions of meshes 3 to 5 are in the asymptotic region of convergence ($p_{a}$ represents the formal order of accuracy). Because the relative error $\varepsilon_{mn}$ between mesh 4 and 5 was only 2.3% but the simulation time increased by 39.0%, mesh 4 was used for further investigations. The same investigations were carried out for the shake flask without baffles, again using a mesh with $1.40\cdot 10^{6}$ cells.

The experimental determination of the specific power input in orbitally shaken systems can only be achieved using a very complex setup. However, Büchs et al. [43] carried out experimental investigations by means of torque measurement and determined an empirical derivation of the specific power input [43,83,84]. The power input for shake flasks, both with and without baffles, is independent of the shaking amplitude as long as they are in phase [85]. Thus, for the process parameters used here with a working volume of 160 mL, a shaking rate of 130 rpm, shaking amplitude of 50 mm and specific power input of 82.4 W/$m^{3}$ (Equation in Büchs et al. [43]) and 83.7 W/$m^{3}$ (Equation in [83]) were obtained for the shake flasks without baffles. The shake flask is in phase with a phase number Ph of 5.48≫1.26 and an axial Froude number ${\mathrm{Fr}}_{a}$ of 0.47>0.4 (Equations (10) and (11)) [85]. The volume of liquid in the shake flask corresponds to *V*, *d* corresponds to the maximum inner diameter of the shake flask, and $\eta_{w}$ corresponds to the dynamic viscosity of the water phase.

(10) $$\mathrm{Ph}=\frac{d_{0}}{d}\left(1-3\;\cdot \;\log\left(\frac{\rho_{w}\;\cdot \;n\;\cdot \;d^{2}}{\eta_{w}}\;\cdot \;\frac{\pi }{2}{\left(1-\sqrt{1-\frac{4}{\pi }{\left(\frac{V^{1/3}}{d}\right)}^{2}}\right)}^{2}\right)\right)$$

(11) $${\mathrm{Fr}}_{a}=\frac{\left(2\;\cdot \;\pi \;\cdot \;d_{0}\right)}{2\;\cdot \;|\vec{g}|}$$

By means of CFD investigations, it was possible to determine the specific power input P/V via the torque *M* acting on the shake flask. Thereby, it is shown that slightly higher values are predicted by Büchs et al. [83] than by the CFD simulation. A power input of 73.7 W/$m^{3}$ (averaged over a shaking period) is predicted by CFD via the determined torque (Equation (12)).

(12) $$P/V=\frac{2\;\cdot \;\pi \;\cdot \;n\;\cdot \;M}{V}$$

The specific power input varies between 73.2 W/$m^{3}$ and 74.2 W/$m^{3}$ over one shaking period. It should be noted, however, that the measured and calculated values of Büchs et al. [83] scatter significantly (>30%), especially with low specific power inputs. In addition, Büchs et al. [83] used glass flasks for the investigations, and in the CFD simulations, contact angles were used that correspond to those of polycarbonate ($\theta_{\mathrm{PC}}$ = 83${}^{\circ }$, $\theta_{\mathrm{glass}}$ = 0${}^{\circ }$ to 26${}^{\circ }$) [55]. As described in Seidel et al. [50], the specific power input is dependent on the contact angle and increases with increasing contact angle. For shake flasks with baffles, there is no empirical formula that can be used for validation. However, Peter et al. [85] described that as long as the shake flasks are in phase, the power input is significantly higher than for shake flasks without baffles under the same process conditions. For shake flasks with baffles, there is also no formula for determining the phase number [85,86,87]. The power input determined by CFD averages 197.4 W/$m^{3}$ over a shaking period and fluctuates between 165.6 W/$m^{3}$ and 239.2 W/$m^{3}$. Due to the non-rotation symmetrical geometry, the power input fluctuates significantly more over the rotation period than with the shake flask without baffles. This simulation confirmed the statements of Peter et al. [85] and Li et al. [88] where the specific power input of shake flasks with baffles is significantly higher than that of shake flasks without baffles. The specific power input can be determined using CFD not only via the torque but also via the energy dissipation rate ε (sum of turbulent and viscous energy dissipation rate) [89]. Because an unstructured mesh was used, where not all mesh cells have the same volume, the local energy dissipation rates $\varepsilon_{i}$ must be multiplied by the corresponding control volume $V_{i}$ (and the density of the fluid $\rho_{w}$) and divided by the total fluid volume *V* (Equation (13)). However, the local energy dissipation rate cannot be directly determined from the simulations carried out as the *k*-ω-SST turbulence model was used. However, the local energy dissipation rate $\varepsilon_{i}$ corresponds to the product of local turbulent kinetic energy $k_{i}$, local specific dissipation rate $\omega_{i}$, and model constant $\beta^{*}=0.09$ (Equation (14)).

(13) $$P/V=\frac{\sum \varepsilon_{i}\;\cdot \;V_{i}\;\cdot \;\rho_{w}}{V}$$

(14) $$\varepsilon_{i}=k_{i}\;\cdot \;\omega_{i}\;\cdot \;\beta^{*}$$

In this case, a specific power input of only 46.7 W/$m^{3}$ instead of 73.7 W/$m^{3}$ is determined for the shake flask without baffles. This underestimation of the specific power input is typical for the approach that utilises the energy dissipation rate. Multiple authors have shown that the power input determined by this method is up to 50% lower than that determined via torque [90,91,92,93]. Tianzhong et al. [94] described the ratio of specific power input determined via torque to volume-averaged energy dissipation rate ($\bar{\varepsilon }\;\cdot \;\rho_{w}=0.629\;\cdot \;P/V$), which shows a linear dependence that corresponds to 0.629. This ratio practically corresponds to that of these simulations, where the ratio was 0.633 for the shake flasks without baffles and 0.616 for those with baffles.

**Figure 4:**
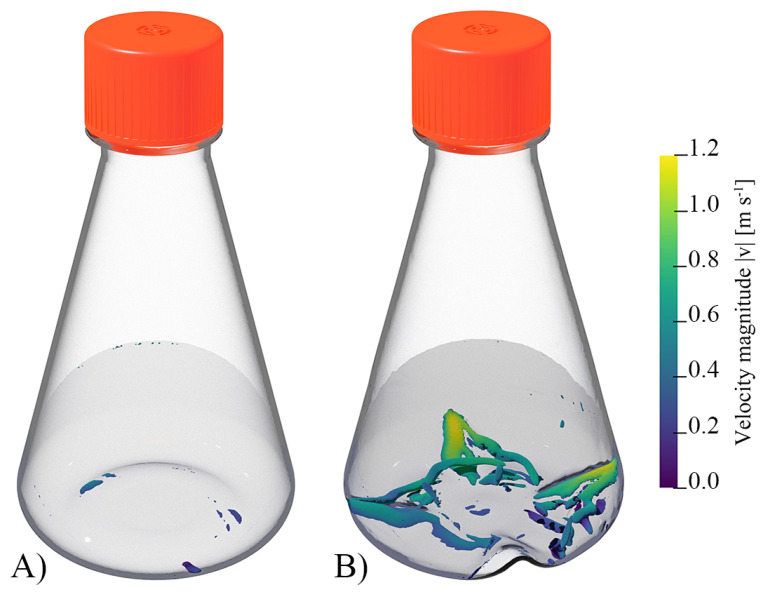
CGI of the CFD simulations for the two shake flask configurations. (**A**) represents the 500 mL shake flask without baffles and (**B**) represents the shake flask with baffles. Shaded in grey is the liquid surface at 130 rpm and 160 mL working volume. Furthermore, the iso-contours at $Q=1000\;s^{2}$ are shown and coloured with the fluid velocity. The iso-contours visualise regions with vortex formation.

Orbitally shaken systems are characterised by their low hydrodynamic heterogeneity $\Phi =\varepsilon_{\max}/\bar{\varepsilon }$, whereby $\varepsilon_{\max}$ is the spacial maximum energy dissipation rate [40,95]. Liu et al. [96] investigated this for both shake flasks with and without baffles using CFD. Thereby, the hydrodynamic heterogeneity for the unbaffled flasks was between 12.75 and 15.87 and between 10.93 and 18.82 for the baffled flasks. Peter et al. [97] experimentally investigated the hydrodynamic heterogeneity in baffled and unbaffled shake flasks by determining the maximum stable droplet diameter, whereby values of up to about 15 were obtained, with the majority of the investigations showing values between 1 and 6. The hydrodynamic heterogeneities determined in this work are consistent with those of Liu et al.’s [96] work and tend to be minimally higher than the values of Peter et al. [97]. The local Kolmogorov length $\lambda_{k,i}$ is directly dependent on the local energy dissipation rate (Equation (15)). In order to calculate a volume-averaged Kolmogorov length ${\bar{\lambda }}_{k}$, the sum of the individual local Kolmogorov lengths $\lambda_{k,i}$ multiplied by the control volumes $V_{i}$ is formed analogous to Equation (13) and divided by the liquid volume *V*. This volume-averaged Kolmogorov length is $6.123\cdot 10^{-5}$ m for the shake flasks with baffles and $1.025\cdot 10^{-4}$ m for the shake flasks without baffles, evaluated over one shaking period. Both values are significantly higher than the determined cell diameter of HEK293 cells (Section 3.2). These high values in combination with the low hydrodynamic heterogeneity suggest that the cells are not expected to be damaged by the hydrodynamic stress [38,98,99]. The hydrodynamic difference between the two shake flasks investigated can be illustrated by means of a vortex visualisation. For this purpose, the widely used *Q*-criterion was used, which corresponds to the second invariant of the velocity gradient tensor (Equation (16)) [100,101,102]. Positive *Q* values correspond to areas where vorticity dominates over viscous stress [100]. Figure 4 shows the two shake flasks at the same time step with the liquid indicated by shading. A value of 1000 s${}^{2}$ was used as the *Q*-criterion. This shows that, compared with the shake flask with baffles, the shake flask without baffles has almost no areas with $Q>1000\;s^{2}$. In the case of the shake flask with baffles, it can be seen that vortex regions form around the four baffles. The fluid velocity is at its maximum near the wall on the ridge of the baffles.

(15) $$\lambda_{k,i}={\left(\frac{\nu^{3}}{\varepsilon_{i}}\right)}^{\frac{1}{4}}$$

(16) $$Q=\frac{1}{2}\left({\left(tr\left(\nabla \vec{v}\right)\right)}^{2}-tr\left(\nabla \vec{v}\;\cdot \;\nabla \vec{v}\right)\right)$$

### 3.2. Cultivations in Shake Flasks

The cultivations in the shake flasks lasted for 192 h. Similar to the cultivations in the Minifors 2, peak cell density was reached after a cultivation time of 120 h, with a maximum VCD of $(5.57\pm 1.17)\cdot 10^{6}$ cells m$L^{-1}$ in the baffled shake flasks. The maximum VCD in the unbaffled shake flasks was $(4.59\pm 0.45)\cdot 10^{6}$ cells m$L^{-1}$. In the shake flasks without baffles, a metabolism shift took place. HEK293 cells metabolise lactate and glucose concomitantly under certain environmental conditions. Martínez-Monge et al. [103] describe that when lactate and extracellular proteins accumulate, cells start consuming lactate and glucose concomitantly. The cells started to metabolise lactate after a cultivation time of 120 h. At this time, the glucose concentration was 1.92 g/L. The glucose and lactate concentrations at the end of the process were 1.16 g/L and 0.71 g/L. In the shake flasks with baffles, the lactate concentration decreased from 1.56 g/L to 1.23 g/L between t=120 h and t=144 h. However, the lactate concentration increased to 1.65 g/L at the end of the cultivation.

Comparing the maximum VCDs between the two shake flask configurations shows that the VCD${}_{\max}$ of $(5.57\pm 1.17)\cdot 10^{6}$ cells m$L^{-1}$ for shake flasks with baffles is higher than that of the one for shake flasks without baffles ( $(4.59\pm 0.45)\cdot 10^{6}$ cells m$L^{-1}$). The null hypothesis $H_{0}$ of a normal distribution of the VCD${}_{\max}$ values could not be rejected by the Shapiro–Wilk test for both configurations (significance level $\alpha_{s}=0.05$) [104]. The homoscedasticity tests (Levene and Bartlett) were also unable to reject the $H_{0}$ hypothesis of homoscedasticity (Table 3) [105,106]. The Student’s *t*-test with the $H_{0}$ hypothesis $\mu_{VCD_{\max,\;\mathrm{unbaffled}}}=\mu_{VCD_{\max,\;\mathrm{baffled}}}$ shows that there is a statistically significant difference between the two configurations in terms of maximum VCD ($p_{s}=0.003$, $\alpha_{s}=0.05$). To ensure that the statistically significant difference in mean VCD${}_{\max}$ does not result from an oxygen limitation in the shake flasks without baffles, the theoretical maximum cell density up to an oxygen limitation was determined. For this purpose, the formula for maximum oxygen transfer rate ${\mathrm{OTR}}_{\max}$ described by Meier et al. [107] was used. The measured osmolality of the FreeStyle^TM^ 293 medium was $(271.67\pm 0.58)\mathrm{mOsm}\;{\mathrm{kg}}^{-1}$. If oxygen transfer rate (OTR) and oxygen uptake rate (OUR) are in equilibrium, the specific oxygen uptake rate $q_{O_{2}}$ can be used to determine the theoretical maximum VCD (Equations (17) and (18)) [108]. $c_{O_{2}}^{*}$ corresponds to the dissolved oxygen concentration at the gas–liquid interphase, $c_{O_{2}}$ to the dissolved oxygen concentration in the liquid bulk, and $c_{x}$ to the biomass concentration.

The maximum specific oxygen uptake rate of $1.85\cdot 10^{-13}$ mol $h^{-1}$ ${\mathrm{cells}}^{-1}$ for HEK293 described in the literature was used as the specific oxygen uptake rate [109,110]. In order to calculate the theoretical solubility of oxygen, the simplified assumption was made that oxygen was dissolved in water and calculated according to Pappenreiter et al. [111] and Tromans [112]. Thus, a volumetric oxygen mass transfer coefficient $k_{L}a$ value of 16 $h^{-1}$ results in a theoretical oxygen supplementation that is suitable for a cell density of $14\cdot 10^{6}$ cells m$L^{-1}$, which is significantly higher than the cell density reached in all experiments. This calculation is only an approximation, and phenomena such as the biological enhancement factor and other limitations have not been taken into account [113].

(17) $$\mathrm{OTR}=k_{L}a\;\cdot \;\left(c_{O_{2}}^{*}-c_{O_{2}}\right)$$

(18) $$\mathrm{OUR}=c_{x}\;\cdot \;q_{O_{2}}$$

**Figure 5:**
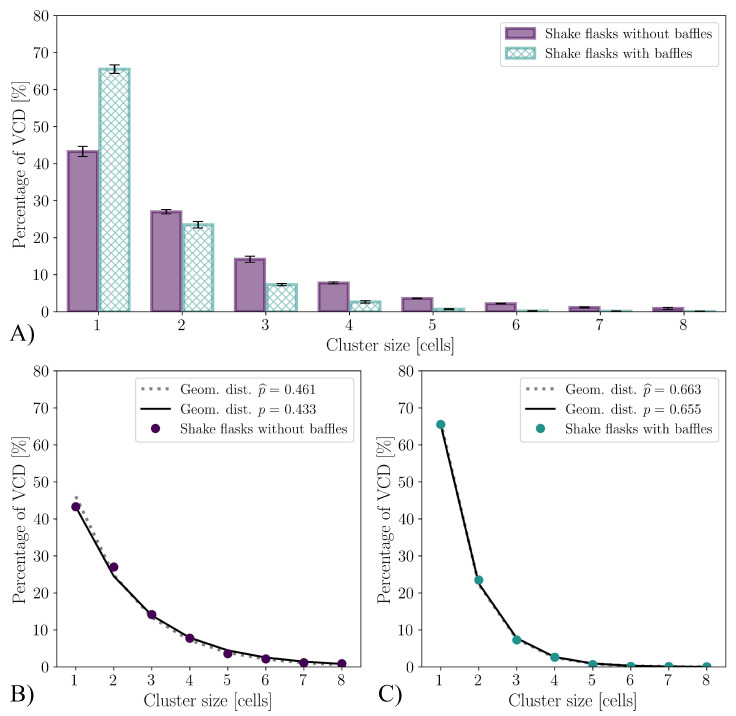
Cluster size distribution at the cultivation time, at which the VCD is maximum $t_{VCD_{\max}}$. (**A**) Comparison of the cluster size distribution for the cultivations with the two different shake flask configurations. (**B**,**C**) show the size distribution measured and approximated by the geometric distribution. The geometric distribution where parameter *p* is equal to the proportion of non-aggregated viable cells is shown as a solid line. The dashed line, in contrast, shows the geometric distribution with parameter *p* that is determined by the maximum likelihood estimation $\hat{p}$.

A significant difference is visible in the aggregate size distribution. As can be seen in Figure 5A, in the cultivations using shake flasks with baffles, an average of 65.0% of the viable cells are present as single cells at the time of reaching the maximum VCD. This is significantly more than the 43.3% in the cultivations of the shake flasks without baffles. In the literature, aggregate size distributions are often related to the aggregate diameter [114,115,116,117]. In general, cluster size distributions can be described by a discrete log-normal distribution [118]. Mendes et al. [119] describes the cluster size distribution for monolayers for head and neck cancer-5 (HN-5), human epithelioma-2 (HEp-2) and Madin–Darby canine kidney (MDCK) cells using power law distribution. However, this has the disadvantage of the model parameters having to be determined for each distribution. Figure 5B,C show that the cluster size distribution for the shake flasks both with and without baffles follow a geometric distribution (Equation (19)). The parameter *p* describing the distribution corresponds to the fraction of cells that are not present as aggregates, with *n* corresponding to the aggregate size. Thus, *p* corresponds to 0.433 for the shake flasks without baffles and p=0.655 for those with baffles. If the maximum likelihood estimation $\hat{p}$ for the available data is used instead of the fraction of non-aggregated cells, $\hat{p}$ would be $\hat{p}=0.461$ and 0.663, respectively. The relative differences of the parameter *p* are 6% for shake flasks without baffles and 1% for shake flasks with baffles.

(19) $$f\left(n\right)={\left(1-p\right)}^{n-1}p\;,\left\{p|0\leq p\leq 1\right\}$$

There are several statistical tests to investigate the goodness-of-fit for geometric distributions [120,121,122]. If the widely used $\chi^{2}$ test is used to determine the goodness-of-fit, ($H_{0}$: cluster size distribution is geometrically distributed), it is shown with *p*-value $p_{s}<0.001$ that the cluster size distribution is statistically significantly different from a geometric distribution with p=0.433 ($\alpha_{s}=0.05$, number of HEK293 cells $n_{\mathrm{HEK}}=8190$, $\chi^{2}=160.6$). The same can be observed with the G test (log-likelihood-ratio) and for the shake flasks with baffles ($p_{s}\ll 0.001$, $\alpha_{s}=0.05$, $n_{\mathrm{HEK}}=6912$ $\chi^{2}=71$). The detection of a statistically significant difference can be expected with such a large sample number because the statistical power of the test is extremely high [123,124]. Due to the high sample size, comparing the cluster size distributions predicted by the geometric distribution with the measured ones shows a statistically significant difference, but this has no practical relevance (Figure 5B,C).

**Figure 6:**
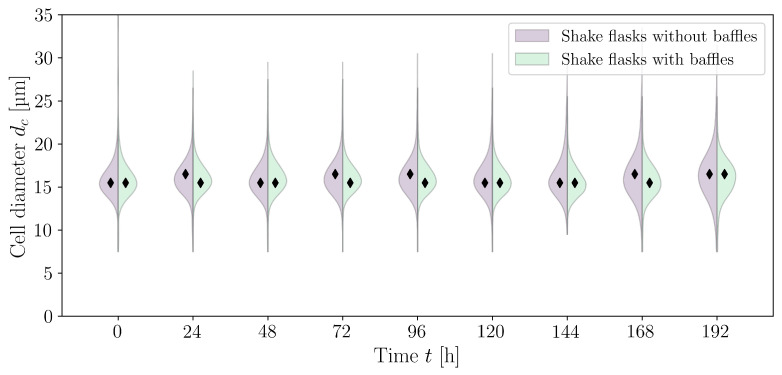
Cell size distribution over time for the shake flask cultivations with ($n_{r}=5$) and without baffles ($n_{r}=5$). Black diamonds (⧫) mark the median cell diameter.

In addition to the aggregate size distribution, the cell size distribution can also be analysed (Figure 6). Maschke et al. [38] describe a normally distributed cell size for CHO XM111-10 with a mean cell diameter of 15.20 μm at the beginning of the exponential growth phase and 14.59 μm at the end. The standard deviation increases from 1.5 μm to 1.7 μm. A normal distribution of cell sizes could also be assumed for the HEK293 cells examined here; however, the quantile–quantile plot showed slightly heavier tails (plots are not shown here). Furthermore, it should be noted that the CedexHiRes analyser only allows for the measurement of cell size distribution in 1 μm classes. For shake flasks without baffles, the mean cell diameter increases from 15.97 μm (cultivation time t=0 h, σ=2.79 μm, $n_{\mathrm{HEK}}=1917$) to 16.50 μm (t=192 h, σ=3.29 μm, $n_{\mathrm{HEK}}=17538$). For the cultivations in shake flasks with baffles, the mean cell diameter remained constant (t=0 h, μ=15.97 μm, σ=2.796 μm, $n_{\mathrm{HEK}}=2138$ to t=192 h, μ=15.95 μm, σ=2.34 μm, $n_{\mathrm{HEK}}=36048$). Liu et al. [21] measured cell diameters of 14.29 μm to 16.32 μm for HEK293 cells with the Cedex AS20 cell counter. Dietmair et al. [125] measured a mean cell diameter of (15.5±0.3) μm for HEK293 cells, and Blumlein et al. [126] assumed a diameter of 15 μm. All of the values are within the range of the values measured here.

### 3.3. CFD for Stirred Bioreactor

As for the CFD investigations with the shake flasks, a mesh study was also carried out for the Minifors 2 using the GCI approach. Four meshes and thus two GCI cases were distinguished (Table 4). Again, care was taken to ensure that 1.1≤r≤1.3 and the volume-averaged Kolmogorov length $\bar{\lambda_{k}}$ were used as the GCI criteria. The results in Table 4 show that the quotient $\frac{{\mathrm{GCI}}_{i+2,i+1}}{r^{p_{a}}{\mathrm{GCI}}_{i+1,i}}$ is close to one only for the second case, which indicates an asymptotic approximation. Due to the small relative deviation of $8.43\cdot 10^{-3}$, computational mesh 3 with $4.49\cdot 10^{6}$ cells was used for further investigations.

**Figure 7:**
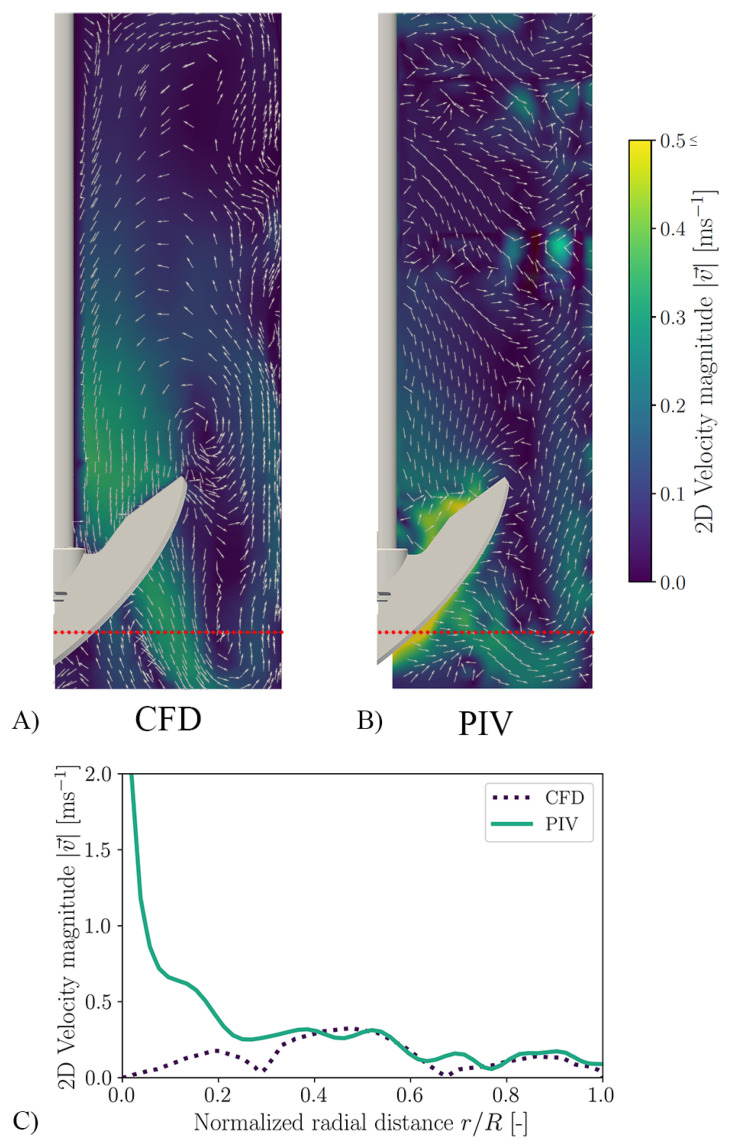
Validation of the CFD simulations for the Minifors 2. (**A**) shows the 2D velocity profile calculated by CFD for a stirrer speed of 180 rpm and working volume of 4 L. (**B**) shows the measured 2D velocity profile using PIV. The shown colour bar applies to both. (**A**–**C**) illustrates the difference between the velocity profiles at a height of 0.05 m from the bottom of the reactor (red line in (**A**,**B**)).

In order to not only quantify the spatial discretisation error but also to validate the model, PIV measurements were performed. Figure 7A shows the 2D velocity field of the CFD simulation at 180 rpm. By using the 2D velocity field, the simulation becomes comparable to the 2D-2C PIV (Figure 7B). Figure 7C shows the velocity profile over the normalised radial distance r/R (at the level of the red line, 0.05 m above the bioreactor bottom; Figure 7A,B). It can be seen that the 2D velocity magnitude between CFD and PIV agrees well. Larger deviations only occur directly at the stirrer. Here, significantly higher velocity magnitudes are predicted by the experiments. However, these high values can be traced back to reflections on the stirrer blade (despite the blades being sprayed black). Another indicator is that these values of 2 m/s are higher than the theoretical maximum speed of 0.80 m/s (stirrer tip speed). A further deviation between PIV and CFD can be observed in the upper area near the wall. The reason for the higher velocity magnitudes in the PIV evaluation is that there is a probe at this point behind the measuring plane, which was also sprayed black, but still resulted in reflections at these two points, which affected the measurement. Despite this deviation, which is largely due to the experiment, the CFD model can be considered validated.

**Figure 8:**
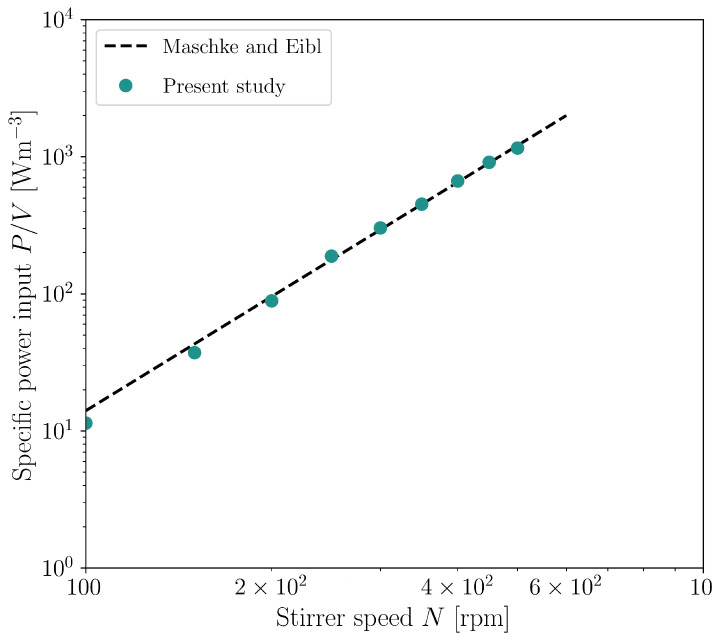
Calculated specific power input for the Minifors 2 with a 4 L working volume. For comparison, the calculations of Maschke and Eibl [127] were used, which describe the specific power input as a function of the tip speed $v_{\mathrm{tip}}$ for a working volume of 4 L ($P/V=131.79\;\cdot \;v_{\mathrm{tip}}^{2.7670}$, $d_{s}=85\;mm$).

If the validated CFD model is used to determine the specific power input, the characteristic pattern for stirred reactors appears, namely, the specific power input being a power function of the stirrer speed [42]. For the Minifors 2, only power inputs are published, which were also determined using CFD [127]. As shown in Figure 8, the specific power inputs determined here correspond to those from the literature [127]. The specific power input increases from 11.4 W/$m^{3}$ (*N* = 100 rpm, Rem=13474) to 1155.3 W/$m^{3}$ (*N* = 500 rpm, Rem=67371). The modified Reynolds number Rem is defined according to Equation (20), with $d_{s}$ representing the stirrer diameter.

(20) Rem=ρw·N·ds2ηw

Maschke and Eibl [127] describe the specific power input for a working volume of 4 L with $P/V=131.79\;\cdot \;v_{\mathrm{tip}}^{2.7670}$, with $v_{\mathrm{tip}}$ corresponding to the tip speed. The calculated Newton number Ne (also known as the power number) decreases from 2.23 at 100 rpm (Rem=13,474) to 1.80 at 500 rpm (Rem=67,371). The Ne number is calculated according to Equation (21). The fact that there is no stagnation of the Ne number as the modified Rem number increases is consistent with the expected behaviour of flows being in the turbulent transition region [128]. Zhu et al. [129] were able to experimentally describe an unaerated Ne number of about 1.7 for a system with a three-blade elephant ear impeller in a stirred bioreactor with a 2.65 L working volume (up-pumping direction). Rotondi et al. [130] were able to show in the Ambr 250 bioreactor (Sartorius AG, Göttingen, Germany) that, depending on the size and angle of the stirrer blades, the Ne numbers lie between 0.61 and 2.07 for the elephant ear impeller. The elephant ear impeller examined here lies in the range described by Rotondi et al. [130].

(21) $$\mathrm{Ne}=\frac{P}{\rho_{w}\;\cdot \;N^{3}\;\cdot \;d_{s}^{5}}$$

**Figure 9:**
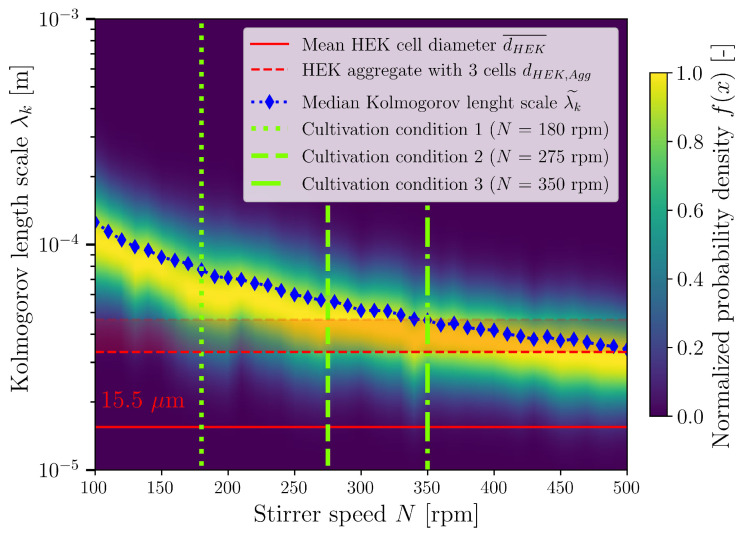
Normalised volume-related probability density function of the Kolmogorov length as a function of the stirrer speed. The respective median of the Kolmogorov lengths is shown as blue diamonds. The three stirrer speeds at which cultivation took place are shown in green as dotted (180 rpm), dashed (275 rpm), and dash-dot (350 rpm) lines. The mean measured HEK293 cell diameter (15.5 μm) is shown as a red line. The size of a three-HEK293 cell cluster is shown as an example with red dashed lines. The lowest value (33.4 μm) corresponds to the close-packing and the highest value (46.5 μm) to a cell chain.

In contrast to orbitally shaken bioreactor systems, stirred bioreactors are characterised by their high hydrodynamic heterogeneity. Depending on the stirrer used, this lies between ≈12 and 400 [131,132,133,134]. For the Minifors 2, a hydrodynamic heterogeneity of 72.4±1.6 was determined. If instead of the energy dissipation rate the dependent Kolmogorov length is represented as a volume-related probability density function, the volume fraction can be determined, which has a cell-critical Kolmogorov length. Figure 9 shows the normalised volume-related probability density function of the Kolmogorov length as a function of the stirrer speed. The solid red line shows the determined mean HEK293 cell diameter. It can be seen that in the investigated range of 11.4 W/$m^{3}$ to 1155.3 W/$m^{3}$ (100 rpm to 500 rpm), the critical eddy size based on the Kolmogorov length scale is larger than the mean cell diameter; thus, no damage should appear. However, the aim of the investigations was to increase the hydrodynamic stress and not to use 13 W/$m^{3}$ to 60 W/$m^{3}$, as is typically the case [69,70,71]. By increasing the hydrodynamic stress and decreasing the Kolmogorov length, there was an attempt to shear cell aggregates and thus minimise the typical aggregate formation of HEK293 cells. By integrating the non-normalised frequency density functions of Figure 9, it can be seen that in the cultivations with 180 rpm, the largest volume fraction has a Kolmogorov length that is above the mean HEK293 cell diameter. The Kolmogorov lengths are lower than 15.5 μm in only 0.2 mL of the 4 L working volume. Only a volume fraction of 0.02077 ( 83 mL) have a Kolmogorov length lower than the size of a three-cell cluster (33.4 μm). In the 275 rpm cultivations, a volume fraction of 0.0777 or 311 mL has a Kolmogorov length lower than 33.4 μm (in 1.7 mL, the Kolmogorov lengths are lower than 15.5 μm) and a volume fraction of 0.121 or 484.1 mL in the 350 rpm cultivations (in 1.9 mL, the Kolmogorov lengths are lower than 15.5 μm).

### 3.4. Cultivations in Stirred Bioreactor

**Figure 10:**
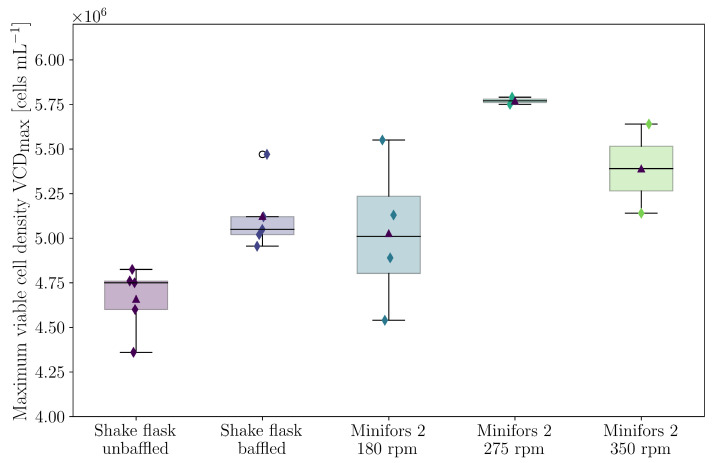
Box plot of the maximum VCD achieved in different cultivation systems and under different process conditions with viability at time ${\mathrm{VCD}}_{\max}$ above 95%.

The batch experiments in the Minifors 2 bioreactor took between 168 h and 196 h. The maximum VCD differed depending on the set stirrer speed and specific power input, respectively (Figure 10). Thus, with a stirrer speed of 180 rpm ($P/V=63\;W/m^{3}$), a VCD of $(5.09\pm 0.36)\cdot 10^{6}$ cells m$L^{-1}$ was achieved. With a stirrer speed of 275 rpm (P/V = 233 W/$m^{3}$), the maximum VCD of $(5.77\pm 0.02)\cdot 10^{6}$ cells m$L^{-1}$ was reached with a speed of 350 rpm ($P/V=451W/m^{3}$) $(5.38\pm 0.26)\cdot 10^{6}$ cells m$L^{-1}$. After about 120 h, the cultivations reached their maximum VCD with viabilities above 95%.

**Figure A4:**
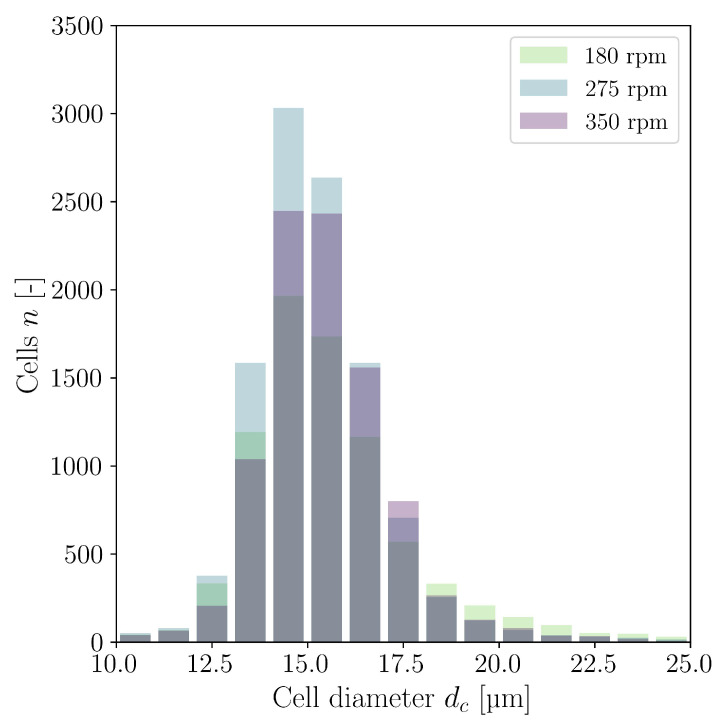
Cell size distribution at the time of maximum VCD measured with the CedexHiRes analyser for different stirrer speeds in the Minifors 2.

The maximum lactate concentrations were between 2.08 g/L and 2.19 g/L for all cultivations and were reached one day before the maximum VCD. The highest lactate concentrations were measured at a speed of 350 rpm. As expected, the lactate concentration decreased again towards the end of the cultivation. Henry et al. [135] determined a specific lactate production rate in the exponential phase of (5.04±0.30) pmol ${\mathrm{cell}}^{-1}d^{-1}$ for HEK293 cells. The increased lactate production of mammalian cells is often associated with increased hydrodynamic stress by some authors. For example, Sorg et al. [136] showed that lactate production in Chinese hamster ovary (CHO) cells increased and product titer decreased when hydrodynamic stress was too high. Liu et al. [137] also showed that the specific lactate production rate in HEK293 cells significantly increased in spinner bioreactors at speeds that were too high. Low shear stress was also shown to stimulate HEK293 productivity [138]. Zhan et al. [138] provided an overview of gene regulation under high and low shear stress. Not all authors were able to demonstrate increased lactate production during increased hydrodynamic stress [139]. However, Godoy-Silva et al. [139] observed a reduction in cell diameter for CHO cells. In the investigations carried out here, neither a significant change in lactate concentration nor a reduction in cell diameter could be observed (Appendix A Figure A4). The average cell diameter at the time of maximum VCD was (15.65±2.53) μm at 180 rpm, (15.28±2.03) μm at 275 rpm and (15.49±1.92) μm at 350 rpm. A further investigation, which was not the aim of this research, could be carried out by analysing the cytoskeleton. It is known that the cytoskeleton rearrangement is a response to non-lethal hydrodynamic stress [140]. For example, actin-binding marker antibodies can be used and studied using immunofluorescence analyses. such investigations have already been carried out for adherent endothelial cells and adherent MDCK cells and could also be carried out for further investigations with the HEK293 suspension cells studied here [141,142].

**Figure 11:**
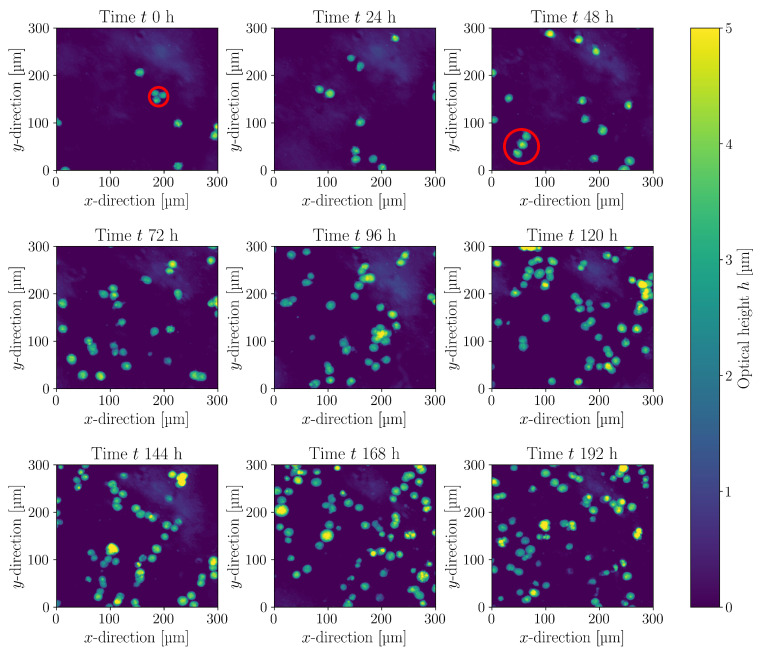
Examples from the evaluation of the optical height at different times of cultivation in the Minifors 2 bioreactor (N=180 rpm, V=4 L). The optical height was determined online using the Ovizio iLine F analyzer. Marked in red are the two extreme forms of a cell cluster with three cells (t=0 h close-packing and t=48 h cell chain).

The maximum specific growth rates $\mu_{\max}$ were achieved in the cultivation period from t= 24 h to 72 h and are comparable for all cultivations. This is (0.0247±0.0017) $h^{-1}$ for 180 rpm, (0.0258±0.004) $h^{-1}$ for 275 rpm, and (0.0250±0.045) $h^{-1}$ for 350 rpm. The values also reflect what is documented in the literature, where typical maximum specific growth rates for HEK293 cells range from 0.020 $h^{-1}$ to 0.029 $h^{-1}$ [23,24,25,26]. In order to follow the aggregate formation and cell morphology online, experiments were carried out using the iLine F probe. Figure 11 shows exemplary sections from the online recording of the holographic images. The optical height *h* of the 3D image was reconstructed using OsOne software through a Fourier transformation [67]. At timepoints t=0 h and t=48 h, two extreme forms of clusters with three cells are marked, which were also used for the assessment of the Kolmogorov length distribution in Section 3.3. Such linear and spherical cell clusters are also described in the literature [143].

**Figure 12:**
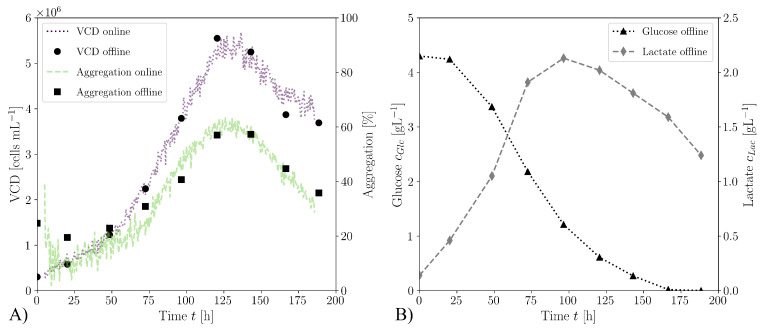
(**A**) Temporal development of VCD and aggregation during cultivation with a stirrer speed of 180rpm. The values measured online with the Ovizio iLine F analyser match the values measured offline over the entire cultivation period. (**B**) Temporal development of the offline measured glucose $c_{Glc}$ and lactate $c_{Lac}$ concentration for the same cultivation.

Figure 12A shows the temporal development of the VCD and aggregation during the cultivation in Minifors 2 at a stirrer speed of 180 rpm. Both the daily offline measured values and those of the online iLineF system are presented. It becomes evident that the values measured offline correspond well with the values measured online over the entire cultivation period. Altenburg et al. [144] showed similar accuracies between the VCD determination using iLineF and offline determination in cultivations with insect cells. The cell aggregation rate correlates with the VCD. Both values increase until the time of t=120 h and then decrease again until the end of the cultivation. The increase in aggregation with increasing cell density is also described in the literature [145]. Figure 12B shows the lactate and glucose concentrations measured offline for the same cultivation.

**Figure 13:**
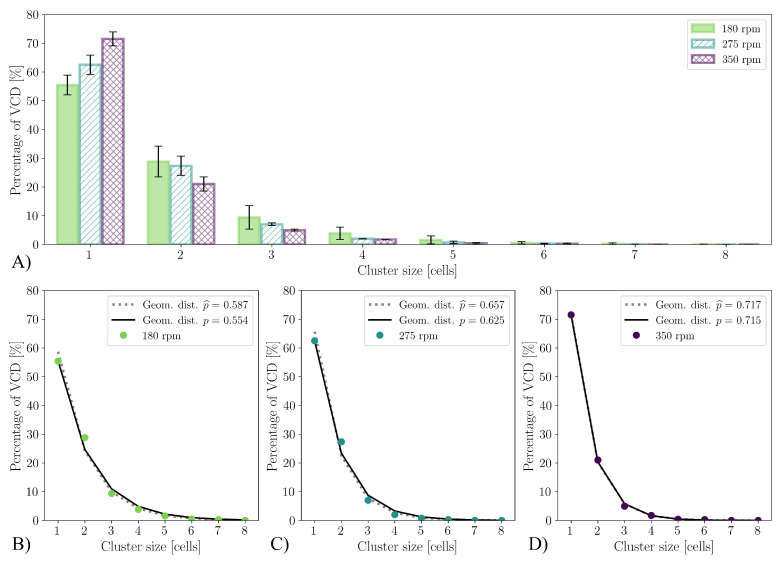
Cluster size distribution at $t_{VCD_{\max}}$. (**A**) comparison of the cluster size distribution at the three investigated stirrer speeds. (**B**–**D**) show the size distribution measured and approximated by the geometric distribution. The geometric distribution where parameter *p* is equal to the proportion of non-aggregated viable cells is shown as a solid line. The dashed line, in contrast, shows the geometric distribution with parameter *p* that is determined by the maximum likelihood estimation $\hat{p}$.

It can be seen that the cell diameter does not change significantly with different specific power inputs, but the aggregation range is strongly dependent on the specific power input. Figure 13 shows the cluster size distribution similar to the experiments with the shake flasks. In Figure 13A, the difference in aggregation at the time of the maximum VCD becomes clearly visible. At a stirrer speed of 350 rpm, only 28.5% of the viable cells are present as aggregates, whereas it is already at 37.5% for a stirrer speed of 275 rpm and 44.6% at 180 rpm. The cluster size distribution also precisely follows a geometric distribution for the cultivations in the stirred bioreactor, as was already the case for the cultivations with the shake flasks. Here, too, the proportion of non-aggregated cells is suitable as a parameter *p* of the geometric distribution. As shown in Figure 13B to Figure 13D, the parameter *p* determined in this way at 350 rpm deviates by only 0.28% from the maximum likelihood estimated parameter $\hat{p}$ (5.12% at 275 rpm and 5.95% at 180 rpm).

**Figure 14:**
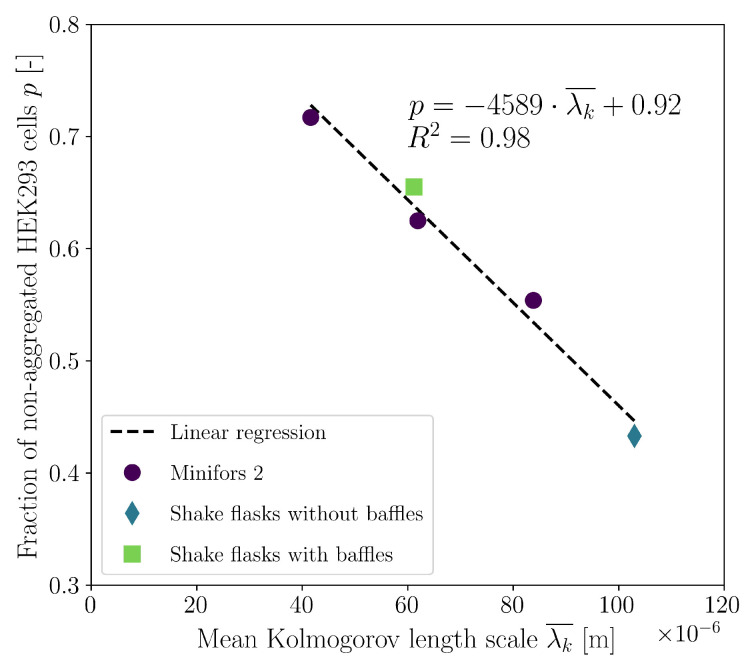
Dependence of the proportion of non-aggregated cells on the mean Kolmogorov length scale. The linear relationship applies independently of the investigated cultivation system and type of mechanical power input. The dependent parameter *p* can be used to predict the aggregate size distribution via the geometric distribution (Equation (19)) at the time of maximum VCD.

When the proportion of non-aggregated cells is expressed as a function of the volume-averaged Kolmogorov length scale, a linear relationship can be observed irrespective of the cultivation system and the type of mechanical power input (Figure 14). This insight now allows for the use of the mean Kolmogorov length scale determined by CFD to predict the aggregate size distribution at the time of maximum VCD. The linear relationship described in Figure 14 can thus be substituted in Equation (19), which reflects the direct relationship between the aggregate size distribution and mean Kolmogorov length scale (Equation (22)).

(22) $$f\left(n\right)={\left(0.08-4589\;\cdot \;\bar{\lambda_{k}}\right)}^{n-1}\left(-4589\;\cdot \;\bar{\lambda_{k}}+0.92\right)$$

## 4. Conclusions

In this study, the influence of the specific power input on the maximum VCD, the cell diameter, and the cluster size distribution of HEK293 cells was investigated. The experiments were carried out in shake flasks with and without baffles and in the Minifors 2 stirred bioreactor. CFD simulations were performed to determine the flow field, specific power input, and hydrodynamic stress. The complex geometry of the shake flasks with baffles was accurately modelled using 3D scanning. In addition to determining the discretisation error, the simulations were validated using 2C-2D PIV and P/V data from the literature. For shake flasks, it was shown that the use of baffles creates more vortex structures and significantly increases the specific power input. Increasing the specific power input from 73.7 W/$m^{3}$ to 197.4 W/$m^{3}$ led to a significant decrease in the fraction of aggregated cells and a statistically significant increase in the maximum VCD from $(4.66\pm 0.17)\cdot 10^{6}$ cells m$L^{-1}$ to $(5.12\pm 0.18)\cdot 10^{6}$ cells m$L^{-1}$ (at a constant mean cell diameter). Similar findings were obtained from the experiments with the stirred bioreactor. The maximum VCD can be increased by increasing the specific power input; for example, $(5.09\pm 0.36)\cdot 10^{6}$ cells m$L^{-1}$ was achieved at a stirrer speed of 180 rpm (which corresponds to 63 W/$m^{3}$ and is frequently used in the literature). If the specific power input was increased to 233 W/$m^{3}$, the maximum VCD increased to $(5.77\pm 0.02)\cdot 10^{6}$ cells m$L^{-1}$. A further increase in the power input ( 451 W/$m^{3}$) led to a further reduction of the cell aggregation, but the maximum VCD also decreased to $(5.38\pm 0.26)\cdot 10^{6}$ cells m$L^{-1}$. The online measurement of VCD and aggregation was consistent with the values measured offline over the entire cultivation period. Regardless of the cultivation system and type of mechanical power input, it was shown that the cluster size distribution strictly follows a geometric distribution in which the free parameter *p* corresponds to the proportion of viable cells that are not present as aggregates. Furthermore, a linear relationship between the mean Kolmogorov length scale and the parameter *p* was found. This allows for the calculation of the mean Kolmogorov length scale using CFD and the prediction of the aggregate size distribution in silico by means of the linear relationship found and geometric distribution. The process engineering investigation demonstrated here makes it possible to optimise bioprocesses with HEK293 cells growing in suspension with regard to their maximum VCD, which is particularly essential for inoculum production but also for the manufacturing of products. The characterisation carried out here is important and serves as a basis for the planned next step of scaling up the HEK293 batch process to a pilot scale using Kolmogorov length distribution.

## Figures and Tables

**Table 2:** Overview of GCI analysis for the baffled shake flask at a shaking rate of 130 rpm and shaking amplitude of 50 mm. The number of mesh cells corresponds to $n_{c}$, the volume-averaged Kolmogorov length scale to $\bar{\lambda_{k}}$, the mesh refinement factor to *r*, the observed order of accuracy to ${\hat{p}}_{a}$, and the relative error to $\varepsilon_{mn}$. M1: $n_{c}=0.28\cdot 10^{6}\mathrm{cells}$, $\bar{\lambda_{k}}=6.906\cdot 10^{-5}\;m$; M2: $n_{c}=0.54\cdot 10^{6}\mathrm{cells}$, $\bar{\lambda_{k}}=6.682\cdot 10^{-5}\;m$; M3: $n_{c}=0.89\cdot 10^{6}\mathrm{cells}$, $\bar{\lambda_{k}}=6.380\cdot 10^{-5}\;m$; M4: $n_{c}=1.40\cdot 10^{6}\mathrm{cells}$, $\bar{\lambda_{k}}=6.123\cdot 10^{-5}\;m$; M5: $n_{c}=2.09\cdot 10^{6}\mathrm{cells}$, $\bar{\lambda_{k}}=5.997\cdot 10^{-5}\;m$.

Case	Mesh	** r **	** ${\hat{p}}_{a}$ **	** $\varepsilon_{\mathrm{mn}}$ **	**GCI [%]**	** $\frac{{\mathrm{GCI}}_{i+2,i+1}}{r^{p_{a}}{\mathrm{GCI}}_{i+1,i}}$ **
Case 1	M1-M2	1.24	1.95	$3.24\cdot 10^{-2}$	7.81	0.36
	M2-M3	1.19		$4.52\cdot 10^{-2}$	14.39	
Case 2	M2-M3	1.19	1.67	$4.52\cdot 10^{-2}$	17.21	0.73
	M3-M4	1.16		$4.03\cdot 10^{-2}$	17.65	
Case 3	M3-M4	1.16	2.35	$4.03\cdot 10^{-2}$	11.88	1.20
	M4-M5	1.14		$2.06\cdot 10^{-2}$	6.96	

**Table 3:** Statistical evaluation of the shake flask cultivations, with $n_{r}$ corresponding to the number of cultivation runs and *W*, *L*, $\chi^{2}$, and $t_{s}$ to the test statistics.

Cultivation System	$n_{r}$	** $\bar{{\mathrm{VCD}}_{\max}}$ **	** σ **	Shapiro–Wilk Test		Levene Test		Bartlett Test		Student’s *t*-Test	
	[-]	[ cells m$L^{-1}$]	[ cells m$L^{-1}$]	*W*	$p_{s}$	*L*	$p_{s}$	$\chi^{2}$	$p_{s}$	$t_{s}$	$p_{s}$
Unbaffled	5	$4.59\;\cdot \;10^{6}$	$0.45\;\cdot \;10^{6}$	0.870	0.816	0.000	0.985	0.026	0.873	3.767	0.003
shake flask											
Baffled	5	$5.57\;\cdot \;10^{6}$	$1.17\;\cdot \;10^{6}$	0.267	0.108						
shake flask											

**Table 4:** Overview of GCI analysis for the Minifors 2 stirred bioreactor with a stirrer speed of 180rpm. M1: $n_{c}=1.23\cdot 10^{6}\mathrm{cells}$, $\bar{\lambda_{k}}=1.022\cdot 10^{-4}\;m$; M2: $n_{c}=2.50\cdot 10^{6}\mathrm{cells}$, $\bar{\lambda_{k}}=0.980\cdot 10^{-4}\;m$; M3: $n_{c}=4.49\cdot 10^{6}\mathrm{cells}$, $\bar{\lambda_{k}}=0.967\cdot 10^{-4}\;m$; M4: $n_{c}=7.24\cdot 10^{6}\mathrm{cells}$, $\bar{\lambda_{k}}=0.959\cdot 10^{-4}\;m$.

Case	Mesh	** r **	** ${\hat{p}}_{a}$ **	** $\varepsilon_{\mathrm{mn}}$ **	**GCI [%]**	** $\frac{{\mathrm{GCI}}_{i+2,i+1}}{r^{p_{a}}{\mathrm{GCI}}_{i+1,i}}$ **
Case 1	M1-M2	1.27	0.31	$41.4\cdot 10^{-3}$	69.01	2.50
	M2-M3	1.20		$12.7\cdot 10^{-3}$	25.72	
Case 2	M2-M3	1.22	1.31	$12.7\cdot 10^{-3}$	5.45	0.93
	M3-M4	1.17		$8.43\cdot 10^{-3}$	4.55	

## Data Availability

Not applicable.

## Abbreviations

The following abbreviations are used in this manuscript:

CAR	Chimeric antigen receptor
CCD	Charge-coupled device
CFD	Computational fluid dynamics
CFL	Courant-Friedrichs-Lewy
CGI	Computer generated image
CHO	Chinese hamster ovary
DAPI	4’,6-Diamidino-2-phenylindol
EMA	European Medicines Agency
FDA	Food and Drug Administration
GCI	Grid convergence index
HEK	Human embryonic kidney
HEp-2	Human epithelioma-2
MDCK	Madin-Darby canine kidney
MRF	Multiple reference frame
NADPH	Reduced nicotinamide adenine dinucleotide phosphate
NADP+	Nicotinamide adenine dinucleotide phosphate
Nd:YAG	Neodymium-doped yttrium aluminum garnet
PISO	Pressure implicit with splitting of operator
PIV	Particle image velocimetry
PLIC	Piece-wise linear interface calculation
PMMA	Poly(methyl methacrylate)
RANS	Reynolds-averaged Navier–Stokes
SIMPLE	Semi-Implicit Method for Pressure Linked Equations
SST	Shear stress transport
VOF	Volume of fluid

## Nomenclature

**Latin symbols**		
$a_{1}$	Model constant in Equation (A1)	[-]
$c_{CO_{2}}$	Concentration of CO${}_{2}$ in the shaking incubator	[%]
$c_{O_{2}}^{*}$	Dissolved oxygen concentration at the gas liquid interphase	[$\mathrm{mol}\;m^{-3}$]
$c_{O_{2}}$	Dissolved oxygen concentration in the liquid bulk	[$\mathrm{mol}\;m^{-3}$]
$c_{x}$	Cell density	[cells m$L^{-1}$]
$CD_{k\omega }$	Positive portion of cross-diffusion in Equation (A3)	[-]
*d*	Maximum inner diameter	[mm]
$d_{c}$	Cell diameter	[μm]
$d_{0}$	Shaking amplitude	[mm]
$d_{s}$	Stirrer diameter	[mm]
DO	Dissolved oxygen concentration	[%]
*f*	Geometric function	[-]
$\vec{F}$	Surface tension force	[N]
$F_{s}$	Safety factor	[-]
$F_{1}$	Blending function in Equation (A3)	[-]
$F_{2}$	Blending function in Equation (A1)	[-]
${\mathrm{Fr}}_{a}$	Axial Froude number	[-]
$\vec{g}$	Gravitational acceleration	[$m/s^{2}$]
*h*	Optical height	[μm]
$H_{0}$	Null hypothesis	[-]
*I*	Second order identity tensor	[-]
*k*	Turbulent kinetic energy	[m${}^{2}$/s${}^{2}$]
$k_{i}$	Local turbulent kinetic energy	[m${}^{2}$/s${}^{2}$]
$k_{L}a$	Volumetric oxygen mass transfer coefficient	[$h^{-1}$]
*L*	Test statistic of the Levene-test	[-]
*M*	Moment/Torque	[Nm]
$\vec{n}$	Surface normal vector	[-]
$\vec{n_{n}}$	Unit vector in normal direction	[-]
$\vec{n_{t}}$	Unit vector in tangential direction	[-]
*N*	Shaking/Stirring speed	[rpm]
*n*	Aggregate/Cluster size	[-]
$n_{c}$	Number of mesh cells	[-]
$n_{\mathrm{HEK}}$	Number of HEK293 cells	[-]
$n_{r}$	Number of cultivation runs	[-]
Ne	Power (Newton) number	[-]
OTR	Oxygen transfer rate	[mol $m^{3}$/s]
OUR	Oxygen uptake rate	[mol $m^{3}$/s]
Ph	Phase number	[-]
*P*	Power	[W]
*p*	Free parameter of the geometric distribution	[-]
$\hat{p}$	Maximum likelihood estimation of *p*	[-]
$p_{a}$	Formal order of accuracy	[-]
${\hat{p}}_{a}$	Observed order of accuracy	[-]
$P_{k}$	Production of turbulent kinetic energy	[$m^{2}$/$s^{3}$]
$p_{p}$	Pressure	[Pa]
$p_{s}$	p-value	[-]
P/V	Specific power input	[W/$m^{3}$]
*Q*	Second invariant of the velocity gradient tensor	[$s^{-3}$]
$q_{O_{2}}$	Cell specific oxygen uptake rate	[$\mathrm{mol}\;{\mathrm{cells}}^{-1}\;s^{-1}$]
*r*	Mesh refinement factor	[-]
$R^{2}$	Coefficient of determination	[-]
Rem	Modified Reynolds number	[-]
RH	Relative humidity	[%]
r/R	Normalized radial distance	[-]
*S*	Vorticity magnitude	[$s^{-1}$]
$S_{ij}$	Reynolds stress tensor	[m${}^{-2}$]
*T*	Temperature	[K]
*t*	Time	[s]
$t_{s}$	Test statistic of the *t*-test	[-]
$t_{{\mathrm{VCD}}_{\max}}$	Time at VCD${}_{\max}$	[h]
TCD	Total cell density	[cells m$L^{-1}$]
*V*	Volume	[$m^{3}$]
$V_{i}$	Control volume	[$m^{3}$]
$\vec{v}$	Velocity	[$m\;s^{-1}$]
$v_{\mathrm{tip}}$	Stirrer tip speed	[m $s^{-1}$]
VCD	Viable cell density	[cells m$L^{-1}$]
VCD${}_{\max}$	Maximum viable cell density	[cells m$L^{-1}$]
*W*	Test statistic of the Shapiro-Wilk-test	[-]
*x*	Spatial coordinate	[m]
*y*	Nearest distance to surface	[m]
**Greek symbols**		
α	Model constant in Equation (A3)	[-]
$\alpha_{i}$	Volume fraction of substance i	[-]
$\alpha_{s}$	Significance level	[-]
β	Model constant in Equation (A3)	[-]
$\beta^{*}$	Constant for the *k*-ω-model	[-]
Δ	Difference	[-]
ε	Energy dissipation rate	[$m^{2}$/$s^{3}$]
$\varepsilon_{\max}$	Spacial maximum energy dissipation rate	[$m^{2}$/$s^{3}$]
$\varepsilon_{i}$	Local energy dissipation rate	[$m^{2}$/$s^{3}$]
$\bar{\varepsilon }$	Volume-averaged energy dissipation rate	[$m^{2}$/$s^{3}$]
$\varepsilon_{mn}$	Relative error	[%]
$\eta_{i}$	Dynamic viscosity of substance i	[Pa s]
$\theta_{i}$	Contact angle of substance i	[${}^{\circ }$]
κ	Local interface curvature	[$m^{-1}$]
$\lambda_{k}$	Kolmogorov length scale	[m]
$\lambda_{k},i$	Local Kolmogorov length scale	[m]
${\bar{\lambda }}_{k}$	Volume-averaged Kolmogorov length scale	[m]
μ	Mean value	[-]
$\mu_{\max}$	Maximum specific growth rate	[$h^{-1}$]
$\nu_{\mathrm{eff}}$	Effective viscosity	[$m^{2}$/s]
$\nu_{i}$	Kinematic viscosity of substance i	[$m^{2}$/s]
$\nu_{T}$	Turbulent eddy viscosity	[$m^{2}$/s]
$\rho_{i}$	Density of substance i	[kg/$m^{3}$]
σ	Standard deviation	[-]
$\sigma_{k}$	Model constant in Equation (A2)	[-]
$\sigma_{\omega }$	Model constant in Equation (A3)	[-]
$\sigma_{\omega 2}$	Model constant in Equations (A3), (A6), and (A7)	[-]
$\sigma_{\mathrm{wa}}$	Surface tension of water and air	[N/m]
$\tau_{ij}$	Turbulent stress tensor	[m${}^{-2}$]
Φ	Hydrodynamic heterogeneity	[-]
ϕ	Generic model constant	[-]
$\phi_{1}$	Generic model constant from *k*-ω-model	[-]
$\phi_{2}$	Generic model constant from *k*-ε-model	[-]
χ	Mixed fluid properties	[-]
$\chi^{2}$	Test statistic of the Bartlett-test	[-]
ω	Specific dissipation rate	[$s^{-1}$]
$\omega_{i}$	Local specific dissipation rate	[$s^{-1}$]
**Indices**		
a	Air	
*i*	Generic index	
*j*	Generic index	
glass	Glass	
pc	Polycarbonate	
PMMA	PMMA	
w	Water	

## Appendix A

In this section, microscope images (Figure A1, Figure A2, Figure A3) are shown to illustrate the shape and size of the HEK FreeStyle^TM^ 293-F cells used and the measurement method employed. Figure A4 shows the cell size distribution of the HEK293-F cells measured in the Minifors 2 at different stirrer speeds and maximum VCD. The average cell diameter at the time of maximum VCD was (15.65±2.53) μm at 180 rpm, (15.28±2.03) μm at 275 rpm, and (15.49±1.92) μm at 350 rpm.

## Appendix B

In this section, the calculation of the turbulent eddy viscosity $\nu_{T}$ (Equation A1) is derived using the *k*-ω-SST model of Menter [45,146]. This equation represents the turbulent eddy viscosity limiter function with the model constant $a_{1}=0.31$, turbulence kinetic energy *k*, specific dissipation rate ω, vorticity magnitude *S*, and blending function $F_{2}$ (Equation (A4)).

(A1) $$\nu_{T}=\frac{a_{1}\;\cdot \;k}{\max(a_{1}\;\cdot \;\omega ,S\;\cdot \;F_{2})}$$

The turbulence kinetic energy *k* and the specific dissipation rate ω are calculated according to Equations (A2) and (A3). *t* represents time, *v* is the velocity, *x* is the spacial coordinates, and $P_{k}$ is the production of turbulent kinetic energy; the kinematic viscosity is ν, the blending function is $F_{1}$ (Equation (A6)), and the model parameters are $\beta^{*}$, $\sigma_{k}$ α, β, and $\sigma_{\omega 2}$.

(A2) $$\frac{\partial k}{\partial t}+v_{j}\frac{\partial k}{\partial x_{j}}=P_{k}-\beta^{*}\;\cdot \;k\;\cdot \;\omega +\frac{\partial }{\partial x_{j}}\left[\left(\nu +\sigma_{k}\;\cdot \;\nu_{T}\right)\frac{\partial k}{\partial x_{j}}\right]$$

(A3) $$\frac{\partial \omega }{\partial t}+v_{j}\frac{\partial \omega }{\partial x_{j}}=\alpha \;\cdot \;S^{2}-\beta \;\cdot \;\omega^{2}+\frac{\partial }{\partial x_{j}}\left[\left(\nu +\sigma_{\omega }\;\cdot \;\nu_{T}\right)\frac{\partial \omega }{\partial x_{j}}\right]+2\left(1-F_{1}\right)\sigma_{\omega 2}\frac{1}{\omega }\frac{\partial k}{\partial x_{i}}\frac{\partial \omega }{\partial x_{i}}$$

In order to solve the equations, additional closure coefficients are necessary. The blending function $F_{2}$ is defined according to Equation (A4), where *y* is the nearest distance to the next surface. Thus, $F_{2}$ becomes larger as the distance to the wall *y* becomes smaller.

(A4) $$F_{2}=\tanh\left\{{\left[\max\left(\frac{2\sqrt{k}}{\beta^{*}\;\cdot \;\omega \;\cdot \;y},\frac{500\;\cdot \;\nu }{y^{2}\;\cdot \;\omega }\right)\right]}^{2}\right\}$$

$P_{k}$ corresponds to the production of turbulent kinetic energy and, in the case of the *k*-ω-SST model, is a limiter function (Equation (A5)). The turbulent stress tensor is represented as $\tau_{ij}$.

(A5) $$P_{k}=\min\left(\tau_{ij}\frac{\partial v_{i}}{\partial x_{j}},10\;\cdot \;\beta^{*}\;\cdot \;k\;\cdot \;\omega \right)$$

Another blending function $F_{1}$ used for the smooth transition between the *k*-ε model ($F_{1}=0$) for regions far from the wall and the *k*-ω model ($F_{1}=1$) for regions close to the wall is defined according to Equation (A6). The term $CD_{k\omega }$ (Equation (A7)) corresponds to the positive portion of the cross-diffusion in Equation (A3).

(A6) $$F_{1}=\tanh\left\{{\left\{\min\left[\max\left(\frac{\sqrt{k}}{\beta^{*}\;\cdot \;\omega \;\cdot \;y},\frac{500\;\cdot \;\nu }{y^{2}\;\cdot \;\omega }\right),\frac{4\;\cdot \;\sigma_{\omega 2}\;\cdot \;k}{CD_{k\omega }\;\cdot \;y^{2}}\right]\right\}}^{4}\right\}$$

(A7) $$CD_{k\omega }=\max\left(2\;\cdot \;\rho \;\cdot \;\sigma_{\omega 2}\frac{1}{\omega }\frac{\partial k}{\partial x_{i}}\frac{\partial \omega }{\partial x_{i}},10^{-20}\right)$$

The empirical constants ϕ required for the model are each determined according to Equation (A8), where $\phi_{1}$ correspond to the model parameters of Wilcox’s [147] *k*-ω model and $\phi_{2}$ corresponds to the parameters of the classical *k*-ε model of Launder and Spalding [148] (Table A1).

(A8) $$\phi =\phi_{1}F_{1}+\phi_{2}\left(1-F_{1}\right)$$

**Table A1:** Overview of the model constants used for the *k*-ω-SST turbulence model.

Coefficient	Value
$\phi_{1}$ from Wilcox [147] (*k*-ω model)	
$\alpha_{1}$	$\frac{5}{9}$
$\beta_{1}$	$\frac{3}{40}$
$\beta^{*}$	$\frac{9}{100}$
$\sigma_{k1}$	$\frac{17}{20}$
$\sigma_{\omega 1}$	$\frac{1}{2}$
$\phi_{2}$ from Launder and Spalding [148] (*k*-ε model)	
$\alpha_{2}$	$\frac{11}{25}$
$\beta_{2}$	$\frac{1}{1250}$
$\beta^{*}$	$\frac{9}{100}$
$\sigma_{k2}$	1
$\sigma_{\omega 2}$	$\frac{107}{125}$
